# MPV17 Loss Causes Deoxynucleotide Insufficiency and Slow DNA Replication in Mitochondria

**DOI:** 10.1371/journal.pgen.1005779

**Published:** 2016-01-13

**Authors:** Ilaria Dalla Rosa, Yolanda Cámara, Romina Durigon, Chloe F. Moss, Sara Vidoni, Gokhan Akman, Lilian Hunt, Mark A. Johnson, Sarah Grocott, Liya Wang, David R. Thorburn, Michio Hirano, Joanna Poulton, Robert W. Taylor, Greg Elgar, Ramon Martí, Peter Voshol, Ian J. Holt, Antonella Spinazzola

**Affiliations:** 1 MRC Mill Hill Laboratory, London, United Kingdom; 2 Laboratory of Mitochondrial Disorders, Vall d’Hebron Institut de Recerca, Universitat Autònoma de Barcelona, Barcelona, Catalonia; 3 Biomedical Network Research Centre on Rare Diseases, Instituto de Salud Carlos III, Madrid, Spain; 4 MRC Mitochondrial Biology Unit, Wellcome Trust-MRC Building, Cambridge, United Kingdom; 5 Mitochondrial Genetics Group, Nuffield Department of Obstetrics and Gynaecology, Women's Centre, The John Radcliffe Hospital, Oxford, United Kingdom; 6 Department of Anatomy, Physiology and Biochemistry, The Swedish University of Agricultural Sciences, Biomedical Center, Uppsala, Sweden; 7 Murdoch Childrens Research Institute and University of Melbourne Department of Paediatrics, Royal Children's Hospital, Flemington Road, Parkville, Victoria, Australia; 8 Department of Neurology, Columbia University Medical Center, New York, New York, United States of America; 9 Wellcome Trust Centre for Mitochondrial Research, Institute of Neuroscience, Newcastle University, The Medical School, Newcastle upon Tyne, United Kingdom; 10 Institute of Metabolic Science, University of Cambridge, Cambridge, United Kingdom; Max Planck Institute for Biology of Ageing, GERMANY

## Abstract

MPV17 is a mitochondrial inner membrane protein whose dysfunction causes mitochondrial DNA abnormalities and disease by an unknown mechanism. Perturbations of deoxynucleoside triphosphate (dNTP) pools are a recognized cause of mitochondrial genomic instability; therefore, we determined DNA copy number and dNTP levels in mitochondria of two models of MPV17 deficiency. In *Mpv17* ablated mice, liver mitochondria showed substantial decreases in the levels of dGTP and dTTP and severe mitochondrial DNA depletion, whereas the dNTP pool was not significantly altered in kidney and brain mitochondria that had near normal levels of DNA. The shortage of mitochondrial dNTPs in *Mpv17*^*-/-*^ liver slows the DNA replication in the organelle, as evidenced by the elevated level of replication intermediates. Quiescent fibroblasts of MPV17-mutant patients recapitulate key features of the primary affected tissue of the *Mpv17*^*-/-*^ mice, displaying virtual absence of the protein, decreased dNTP levels and mitochondrial DNA depletion. Notably, the mitochondrial DNA loss in the patients’ quiescent fibroblasts was prevented and rescued by deoxynucleoside supplementation. Thus, our study establishes dNTP insufficiency in the mitochondria as the cause of mitochondrial DNA depletion in MPV17 deficiency, and identifies deoxynucleoside supplementation as a potential therapeutic strategy for MPV17-related disease. Moreover, changes in the expression of factors involved in mitochondrial deoxynucleotide homeostasis indicate a remodeling of nucleotide metabolism in MPV17 disease models, which suggests mitochondria lacking functional MPV17 have a restricted purine mitochondrial salvage pathway.

## Author Summary

Mitochondrial DNA depletion syndrome (MDS) is a genetically heterogeneous condition characterized by a decrease of mitochondrial DNA (mtDNA) copy number and decreased activities of respiratory chain enzymes. Depletion of mtDNA has been associated with mutations in several genes, which encode either proteins directly involved in mtDNA replication or factors regulating the homeostasis of the mitochondrial deoxynucleotide pool. However, for some genes the mechanism linking mutations and mtDNA depletion is not known. One such gene is *MPV17*, whose loss-of-function causes mtDNA abnormalities in human, mouse and yeast. Here we show that MPV17 dysfunction leads to a shortage of the precursors for DNA synthesis in the mitochondria, slowing DNA replication in the organelle. Not only does mtDNA copy number correlate with dNTP pool size in both mouse tissues and human cells, deoxynucleoside supplementation of the growth medium prevents depletion and restores mtDNA copy number in quiescent MPV17-deficient cells. Hence, our study links MPV17 deficiency, insufficiency of mitochondrial dNTPs, and slow replication in mitochondria to depletion of mtDNA manifesting in the human disease, and places MPV17-related disease firmly in the category of mtDNA disorders caused by deoxynucleotide perturbation. The prevention and reversal of mtDNA loss in MPV17 patient-derived cells identifies potential therapeutic strategy for a currently untreatable disease.

## Introduction

Mitochondria contain their own DNA (mtDNA), which encodes thirteen subunits of the oxidative phosphorylation (OXPHOS) complexes essential for cellular respiration and ATP production. The faithful synthesis and maintenance of mtDNA depends on nuclear-encoded genes which, when mutated, can cause quantitative (depletion) and qualitative (multiple deletions and point mutations) mtDNA abnormalities, resulting in human diseases [1]. MtDNA depletion syndromes (MDS) manifest as severe, tissue-specific diseases of early infancy [2–5] while multiple deletions typically accumulate much later in life, leading to adult-onset phenotypes in skeletal muscle and possibly in brain [6–11]. Although these disorders are genetically heterogeneous, MDS and multiple deletions can result from mutations in the same gene [2–9,11–13]. One such gene is *MPV17*, in which loss-of-function causes a fatal infantile hepatocerebral syndrome with mtDNA depletion [14], or an adult-onset multisystemic disorder with multiple deletions of mtDNA [15,16]. However, neither the function of MPV17 protein nor the mechanism leading to mtDNA perturbation is yet known.

DNA replication in mitochondria, as in the nucleus, depends on a balanced supply of deoxynucleoside triphosphates (dNTPs), the building blocks of DNA. The provision of dNTPs for the mitochondrial replisome is maintained either by *in organello* recycling of the deoxynucleosides or by the import from the cytosol of dNTPs either synthesized *de novo* by ribonucleotide reduction or from the cytosolic deoxynucleoside salvage pathway. In non-cycling cells, DNA replication in the nucleus is suspended, and the production of dNTPs in the cytosol is downregulated; nevertheless, mtDNA replication persists, and a distinct form of cytosolic ribonucleotide reductase (RNR), containing the p53R2 subunit in place of R2, remains active [17–19]. Thus, in non-cycling cells p53R2 plays a critical role in sustaining DNA replication in the mitochondria [17,20], aided by the organelle’s salvage pathway, especially the rate-limiting enzymes, thymidine kinase 2 (TK2) and deoxyguanosine kinase (DGUOK). The importance of dNTP availability for mtDNA integrity is underscored by the number of human diseases caused by mutations in genes encoding factors involved in nucleotide metabolism [2–4,7,21,22]. The first such factor, thymidine phosphorylase (TP), was causally linked to mtDNA abnormalities and a specific mitochondrial disease, MNGIE (mitochondrial neurogastrointestinal encephalomyopathy) in 1999 [7,22]. Deficiency in this catabolic enzyme greatly increases the level of dTTP leading to mtDNA nucleotide substitutions [23], depression of mitochondrial dCTP levels, and mtDNA deletions and depletion [24,25]. Later, other mitochondrial diseases were attributed to genetic inactivation of enzymes involved in the synthesis of dNTPs, namely, TK2 [3], DGUOK [2,3], and p53R2 [4] and recently the GABA transaminase [21], indicating that either a surplus or a deficiency of nucleotide precursors can be detrimental to mtDNA integrity, and thereby cause disease.

Although *in vivo* and *in vitro* models of these disorders are providing invaluable information on the pathways contributing to the cellular dNTP pool, we lack a comprehensive understanding of the factors that serve to maintain the mitochondrial dNTP pool. For example, while mtDNA apparently relies on cytosolic enzymes for dNTP biosynthesis, it has been proposed that these activities and the known salvage pathways in mitochondria are insufficient to support mtDNA replication [26]. Hence, the organelles are inferred to possess the capacity to generate dNTPs *de novo*, and a ribonucleotide reductase activity has been described in mitochondria [27,28] as well as a mitochondrial isoform of dihydrofolate reductase [29]. Transport of dNTPs into the mitochondrion is another area where our knowledge is currently limited. What is clear is that nucleotide pool perturbation is a generic cause of mitochondrial genomic instability, and so we sought to determine the consequences of MPV17 deficiency on dNTP metabolism in murine and human models of the disease. Both models display decreases in the mitochondrial dNTP pool, accompanied by depletion of mtDNA, and highly abundant replication intermediates in liver mitochondria lacking Mpv17. The adverse effects of MPV17 deficiency on mtDNA can be prevented and rescued in cultured cells by deoxynucleoside supplementation, establishing that MPV17 deficiency causes deoxynucleotide insufficiency in mitochondria, which slows replication in the organelle leading to DNA loss. A comparison of the expression of proteins involved in nucleotide metabolism suggests that mitochondrial dNTPs are scarce in MPV17 deficiency owing to repression of the mitochondrial salvage pathway (MSP).

## Results

### Ablation of *Mpv17* causes tissue-specific mtDNA depletion and deficiencies in respiratory chain and ATP synthase complexes

**Fig 1:**
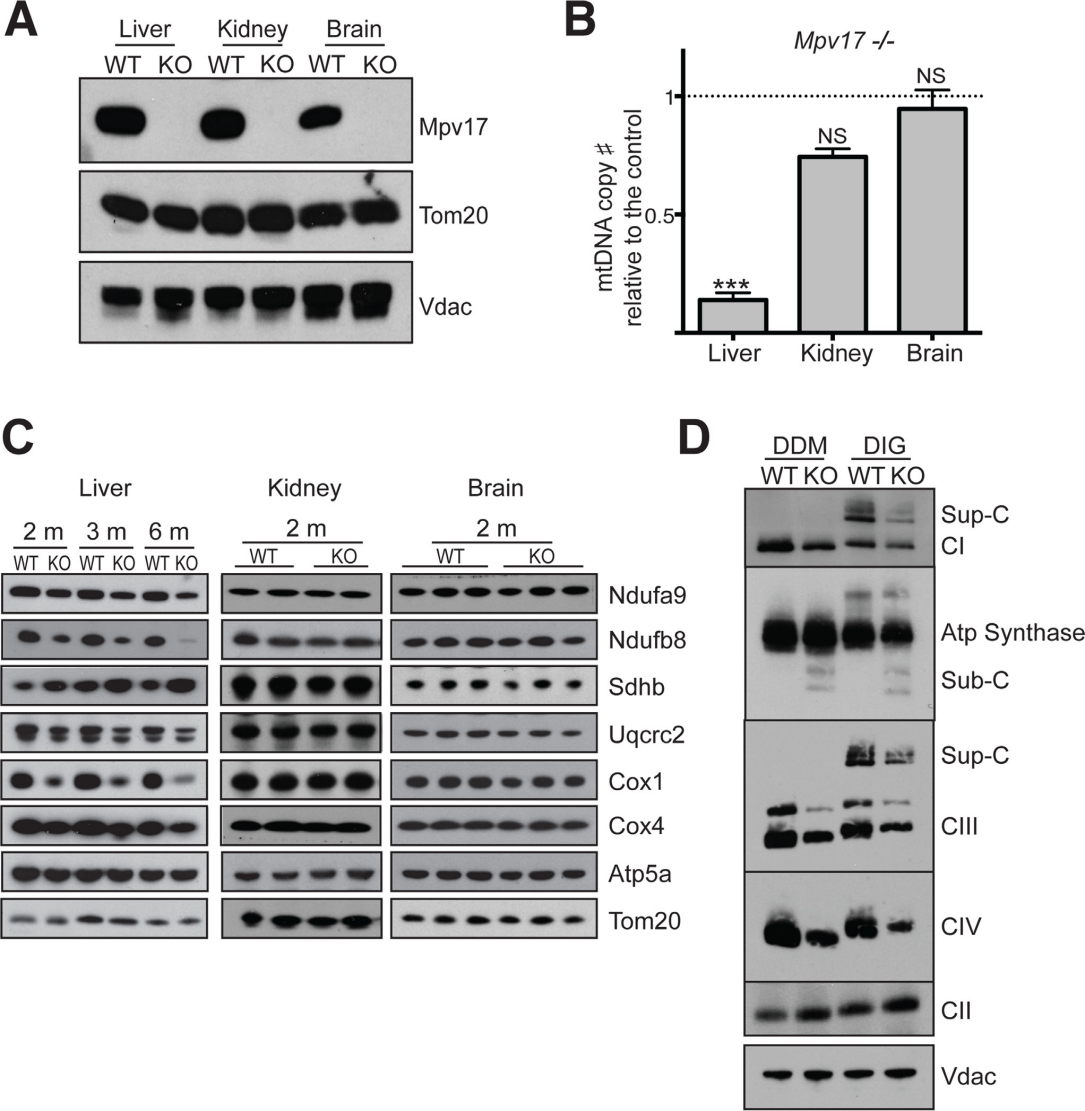
Mpv17 ablation in CFW mice causes liver specific mtDNA depletion and deficiency of all respiratory chain complexes that contain mtDNA-encoded subunits. **(A-C)** Mpv17 expression (**A**), mtDNA copy number (**B**) and steady state levels of OXPHOS subunits (**C**) in the liver, kidney and brain of wild-type (WT, *Mpv17*^*+/+*^*)* and knockout (KO, *Mpv17*^*-/-*^*)* mice. (**B**) Quantification of mtDNA in WT and KO mice. Data are expressed as mean ± SEM of n = 6. (Student test, *** P<0.001, NS, not significant, p>0.05). (**D**) BN-PAGE analysis of OXPHOS complexes in the liver of WT and KO mice. Sup-C, supercomplexes, Sub-C, subcomplexes of the OXPHOS system. Vdac levels were used as a loading control on a 12% SDS-PAGE gel.

The *Mpv17* ablated CFW mouse was used for the study and the genotype was confirmed by PCR analysis (see Methods). Comparison of *Mpv17* ^*-/-*^ mice and wild-type littermates demonstrated the absence of the protein in the liver, brain, and kidney (Fig 1A); however, significant reduction in the level of mtDNA was restricted to the liver (Fig 1B), in mice aged 8–10 weeks. Specifically, mitochondrial DNA copy number in *Mpv17* ^*-/-*^ liver was less than 10% of wild-type mice whereas it was 75% in the kidney and 90% in the brain. The severe depletion of mtDNA in liver was accompanied by decreased steady-state levels of multiple subunits of the OXPHOS complexes (Fig 1C), which were reproduced at the level of the holoenzymes and their supercomplexes (Fig 1D). Furthermore, *Mpv17* ablation was associated with the appearance of sub-complexes of ATP synthase (Fig 1D), another characteristic feature of mtDNA maintenance defects [30]. In contrast, in the kidney and brain of *Mpv17*^*-/-*^ mice, tissues that had no significant decrease in mtDNA copy number, all OXPHOS components analyzed were maintained at levels comparable to wild-type mice (Fig 1C). Another strain of mouse lacking Mpv17 had similar levels of mtDNA in liver, kidney and brain to those reported here [31]. However, the OXPHOS abnormalities of liver mitochondria appear more marked in this study, suggesting that the loss of Mpv17 induces a more severe phenotype on a CFW/MF1, than a C57BL/6 genetic background, as previously reported [31,32].

### *Mpv17* ablated mice have a shortage of the deoxynucleotides dGTP and dTTP in liver mitochondria that slows mtDNA replication

**Fig 2:**
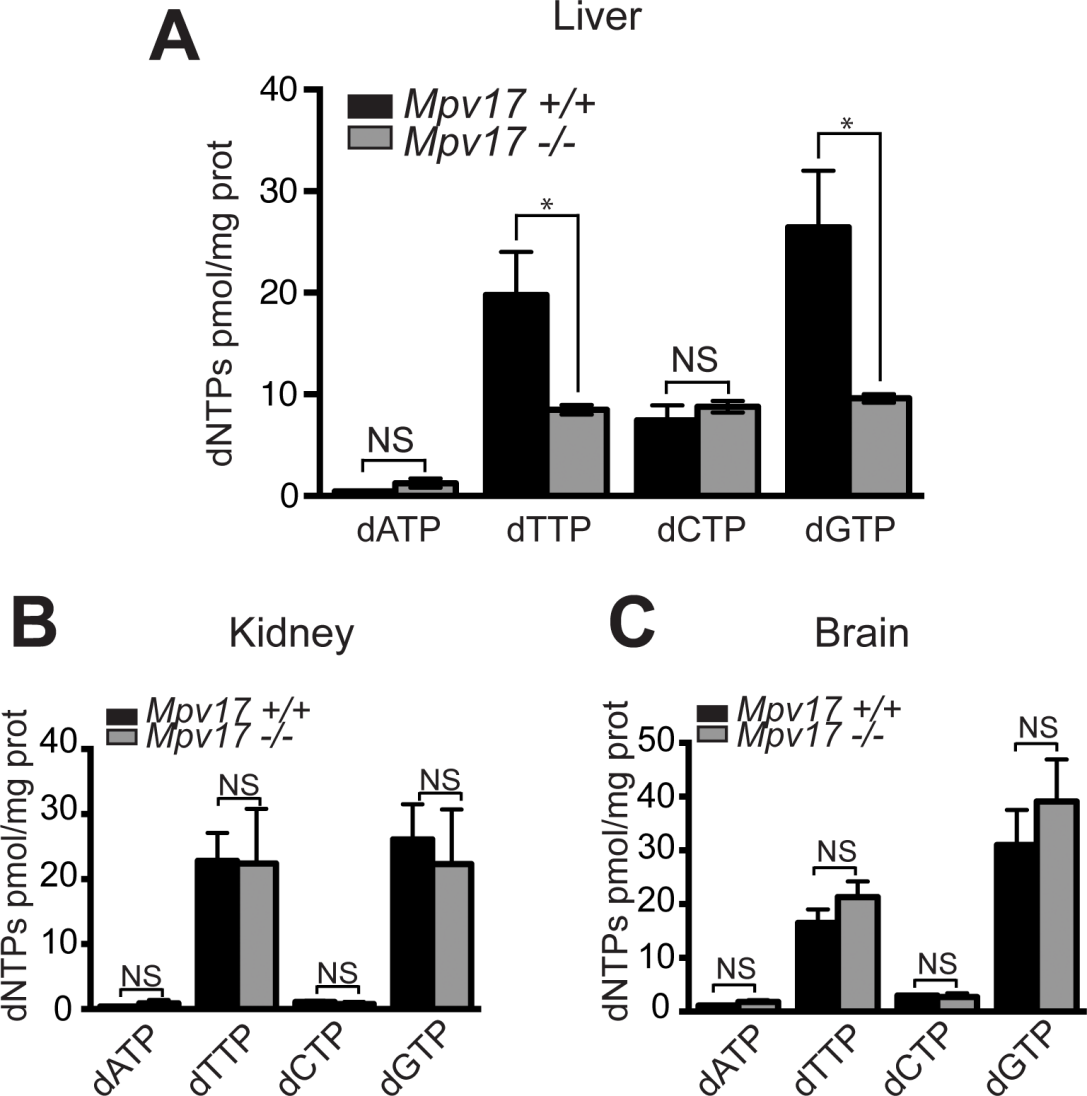
Mpv17^-/-^ mouse liver mitochondria have significantly reduced levels of two precursors of DNA synthesis, dGTP and dTTP. The abundance of dNTPs was determined by means of an extension assay after extraction of nucleotides from liver (**A**), kidney (**B**) or brain (**C**) of *Mpv17*^*+/+*^ and *Mpv17*^*-/-*^ mice (see Methods). All mice (n = 5) were sacrificed between 8 and 10 weeks of age. P values were obtained using Mann-Whitney test (* P<0.05, NS, not significant P>0.05).

As the limited availability of precursors for DNA synthesis is a cause of mitochondrial genomic instability, we determined the levels of mitochondrial dNTPs in the *Mpv17* deficient mouse. Liver mitochondria of *Mpv17*^*-/-*^ mice displayed reduced levels of dGTP (30% relative to the wild-type) and dTTP (35% of wild-type) (Fig 2A), whereas there was no decrease in mitochondrial dNTP levels in kidney or brain (Fig 2B and 2C). Hence a strict correlation exists between mtDNA copy number and dNTP levels in the organelles (Figs 1B and 2), strongly suggesting that mitochondrial nucleotide insufficiency is responsible for the depletion of mtDNA in the liver of the *Mpv17*^*-/-*^ mice.

**Fig 3:**
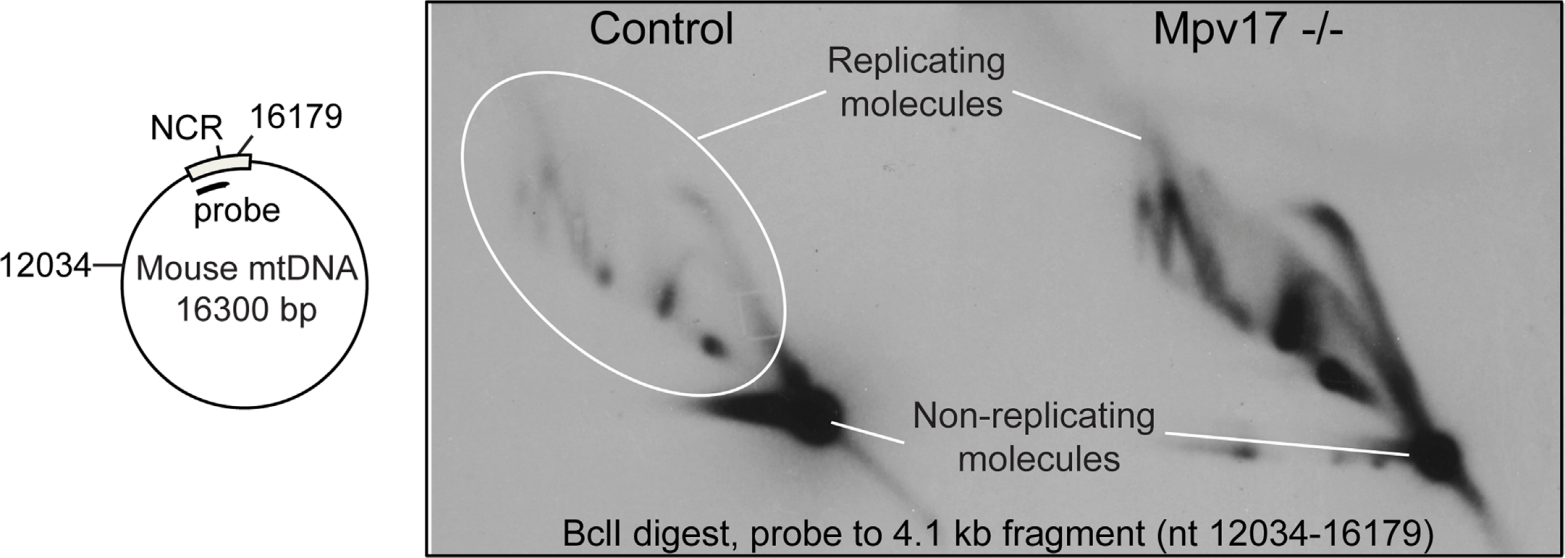
Mpv17 ablation results in a marked increase of mtDNA replication intermediates. Analysis of mtDNA replication intermediates in the liver of WT and *Mpv17*^*-/-*^ mice. Mitochondrial DNAs from six Mpv17^-/-^ and six control livers were isolated, digested with BclI and fractionated by 2D-AGE and blot hybridized to a probe to the major non-coding region (NCR) of the murine mitochondrial genome.

Although nucleotide perturbation was first linked to mitochondrial genomic instability 15 years ago [7,22], the process of mtDNA replication has been little studied in this context [33]. To assess the effect of the nucleotide insufficiency on DNA replication, we analyzed the intermediates of mitochondrial DNA replication of *Mpv17*^*-/-*^ and control liver, using neutral two-dimensional agarose gel electrophoresis (2D-AGE) [34,35]. The striking feature of material isolated from the *Mpv17*^*-/-*^ liver is the high abundance of the replication intermediates (Fig 3, and further interpreted in S1 Fig). This indicates that many more mtDNA molecules are in the process of being replicated in liver mitochondria lacking Mpv17. The fact that the increase in mitochondrial replication intermediates (Fig 3) is associated with mtDNA loss and nucleotide insufficiency (Figs 1B and 2A) strongly suggests that the rate of mtDNA replication is much slower than normal in the liver of the *Mpv17* ablated mouse.

### Quiescent MPV17-deficient human fibroblasts recapitulate the phenotypes of the *Mpv17*^*-/-*^ mouse

**Fig 4:**
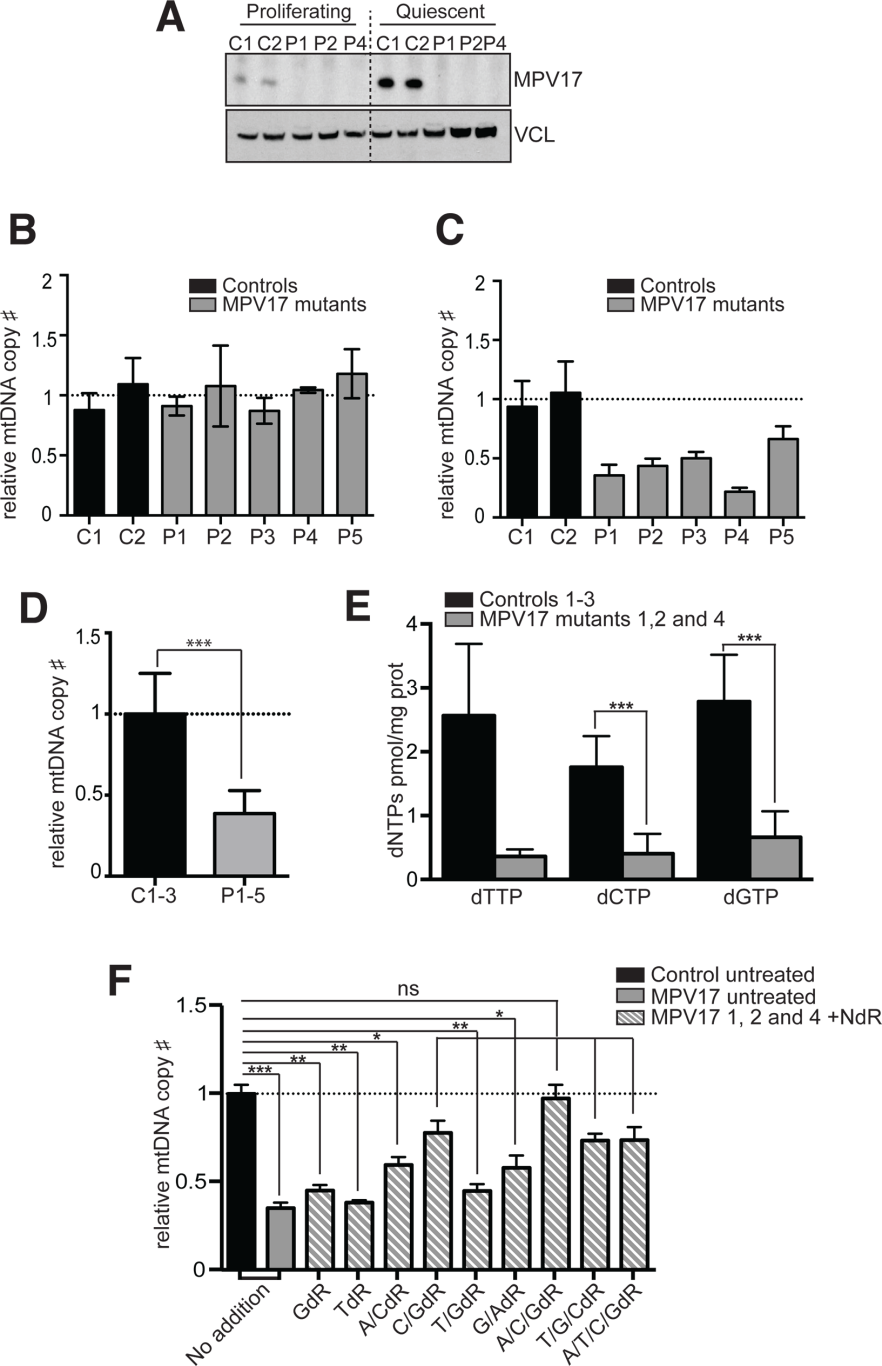
MPV17 is upregulated in non-dividing cells and quiescence induces mtDNA depletion in MPV17-deficient human fibroblasts, unless supplemented with deoxynucleosides. **(A)** Steady state levels of MPV17 protein in proliferating and quiescent control fibroblasts. (**B**) Relative mtDNA levels in proliferating control (black) and MPV1*7* mutant fibroblasts (gray). (**C**) MtDNA quantification in quiescent fibroblasts. The mtDNA amount is expressed relative to the mean of the controls. (**D**) mtDNA copy number of quiescent control and MPV17-mutant fibroblasts. Data are expressed as mean ± SEM of n = 8. (Student’s t test: ***P<0.001). (**E**) Mitochondrial dNTP levels in quiescent control and MPV17-mutant fibroblasts. dATP levels were disregarded owing to the low values obtained for the controls. dCTP and dGTP were measured in 3 independent experiments in 3 patients and 3 control cell lines. *** P<0.001—Mann-Whitney test. (**F)** Relative mtDNA copy number in quiescent fibroblasts supplemented with deoxynucleosides. Quiescent fibroblasts were cultured for 14 days in the absence or presence of 50 μM or 100 μM deoxyadenosine (AdR), deoxycytosine (CdR), deoxyguanosine (GdR), deoxythymidine (TdR) alone or in the different combinations as indicated. The amount of mtDNA is expressed relative to its amount in proliferating cells (Student’s t test: *P<0.05; **P<0.01; ***P<0.001); C1 and C2, control fibroblasts; P1-P5, fibroblasts derived from patients with pathological mutations in *MPV17* (S2 Table).

In proliferating cells mtDNA depends primarily on the dNTP pools derived from cytosolic *de novo* synthesis, and to a minor extent on both the cytosolic and mitochondrial recycling salvage pathways. In non-dividing cells, the cytosolic processes are repressed [36], as evidenced by the marked decreases in expression of the R2 subunit of RNR and thymidine kinase 1 (TK1) (S2A Fig), restricting the dNTPs available for the import into the mitochondria. Therefore, the provision of dNTPs for mtDNA replication relies on the mitochondrial salvage pathway enzymes [37], and on the alternative form of RNR containing the p53R2 subunit [20], which are upregulated in response to quiescence (S2B Fig). Accordingly, proliferating cells from patients with MDS often have normal mtDNA levels, and the gene defects adversely impact mtDNA only when the cells stop dividing [38–40]. Thus, we analyzed the expression of MPV17 protein in proliferating and non-dividing cells and found that it is upregulated in quiescent cells (Figs 4A and S2C), and so closely follows the behavior of other factors linked to MDS that influence mitochondrial dNTP pools (S2B Fig). In five fibroblast cell lines derived from patients with autosomal recessive *MPV17* mutations, all of which displayed decreased expression of the protein (S2D Fig), we found that dividing cells had similar levels of mtDNA to control fibroblasts (Fig 4B), whereas 10–14 days of quiescence led to decreases in mtDNA copy number of 34–80% (Fig 4C). On average the mtDNA copy number in the quiescent MPV17-deficient fibroblasts was 62% lower than the controls (p < 0.001) (Fig 4D). Notably, the smallest decrease in mtDNA copy number among the pediatric cases (patient 5, 34% in fibroblasts, 60% in liver [41]) occurred in the longest surviving child (8.5 years of age at last follow-up). As in the *Mpv17*^*-/-*^ mouse, the loss of mtDNA in human fibroblasts was associated with low dNTP levels, although it appeared to be generalized in the cultured cells, with marked decreases in all three dNTPs that could be quantified (dTTP, dGTP and dCTP) (averaging 80%, relative to controls) (Fig 4E).

### Mitochondrial DNA depletion in quiescent MPV17-deficient fibroblasts can be prevented and rescued by deoxynucleoside supplementation

The low dNTP levels accompanying the depletion of mtDNA (Fig 4C–4E) implied the underlying problem in MPV17 deficiency is a shortage of precursors for DNA synthesis. To further test this hypothesis, we attempted to rescue the mitochondrial dNTP deficiency in *MPV17-*mutant fibroblasts by exploiting the dNTP salvage pathway [20,38,40]. Supplementation of the culture medium with three deoxynucleosides, GdR, AdR and CdR prevented mtDNA depletion in three quiescent MPV17-deficient fibroblast lines (Fig 4F and S3A Fig). Individually, none of the four deoxynucleosides was able to prevent a decrease in mtDNA copy number (Fig 4F), and the same was true of several pairs of deoxynucleosides (A + C, T + G, and G + A); an exception was GdR plus CdR, which was sufficient to prevent significant mtDNA depletion in all three MPV17-deficient fibroblast lines tested (Fig 4F).

**Fig 5:**
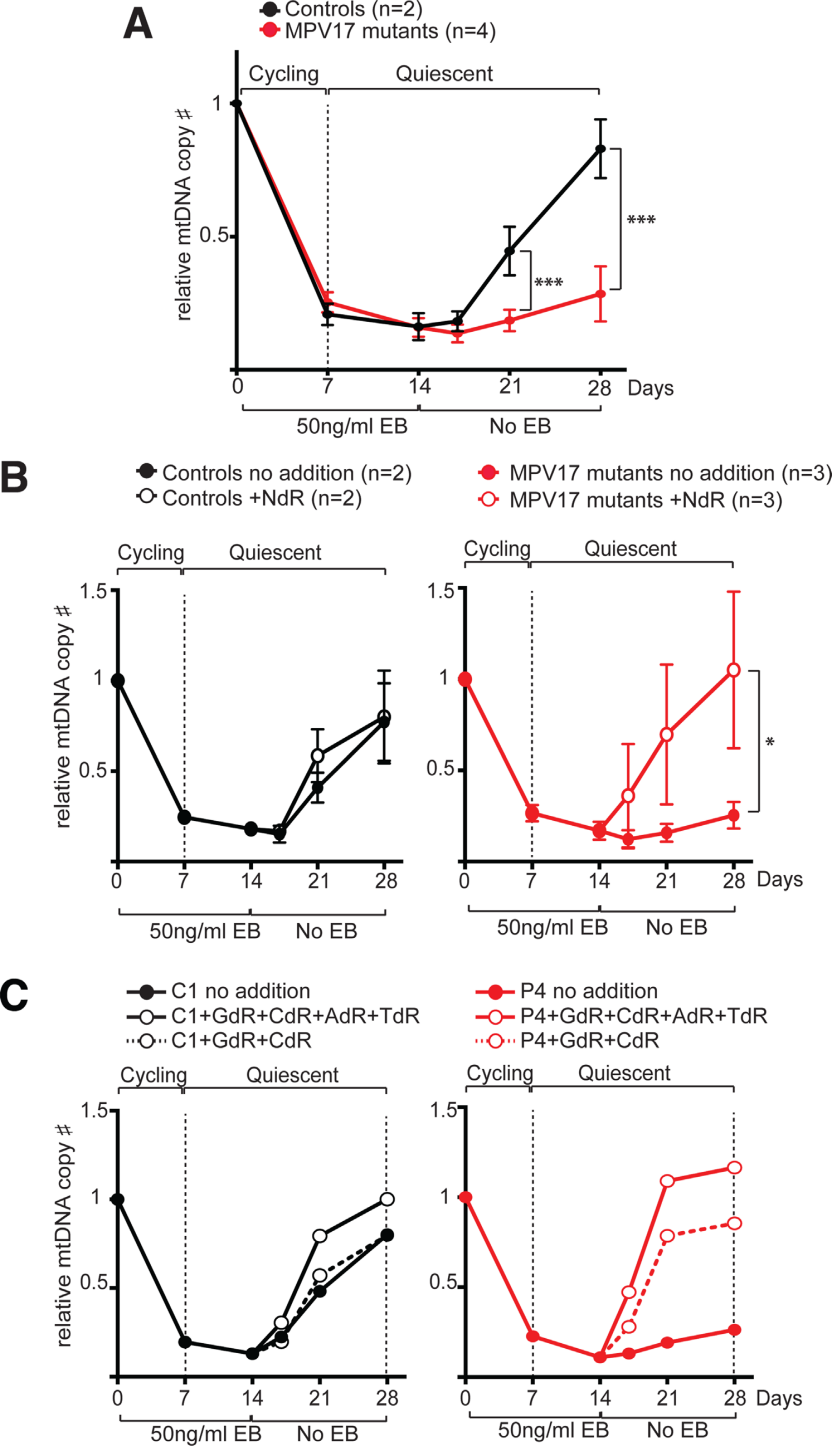
Deoxynucleoside supplementation enables full mtDNA recovery in MPV17-deficient fibroblasts after drug-induced transient depletion. (**A**) Slow recovery of mtDNA copy number in quiescent MPV17-mutant fibroblasts after depletion with ethidium bromide (EB). Two control (black) and four MPV17 patient-derived cell lines (red) were tested in two or three independent experiments. (Student’s t test: ***P<0.001) (**B**) MtDNA recovery in control (black) and MPV1*7*-deficient fibroblasts (red) with or without deoxynucleoside supplementation (open and closed circles, respectively) of 100 μM AdR, CdR, and GdR. Data are expressed as mean ± SEM of two control and three MPV17 patient-derived cell lines, tested in two independent experiments (Student’s *t* test: *P<0.05). (**C**) MtDNA copy number was monitored via Q-PCR during and after ethidium bromide (EB)-induced mtDNA depletion (as per Fig 5B). C1 –control (black) and P4 MPV1*7*-deficient fibroblast (red), with or without (open and closed circles, respectively) 50 μM of the indicated deoxynucleosides.

The experiments in quiescent cells identified nucleoside supplementation as a potential prophylactic treatment for mtDNA depletion in MPV17 deficiency, but did not indicate whether it was beneficial after mtDNA loss has occurred. To investigate whether nucleoside supplementation could rescue, as well as prevent mtDNA depletion in MPV17 deficiency, we performed mtDNA depletion-recovery (repletion) experiments in quiescent cells (see Methods and [20,42]. Repletion was compromised in quiescent MPV17-deficient cell lines: after fourteen days of recovery, the mtDNA level of control cells was close (87%) to the original, whereas in four MPV17-deficient cell lines it was 25% (Fig 5A). This figure increased to 102% in MPV17-deficient cells when the culture medium was supplemented with deoxynucleosides GdR, AdR and CdR, or GdR plus CdR (Fig 5B and 5C). Together these data provide strong evidence that limited precursor availability for DNA synthesis is the underlying cause of the mtDNA depletion in MPV17 deficiency.

### dNTP insufficiency does not alter the mutational load in Mpv17^-/-^ liver mtDNA

MPV17 deficiency has been associated with two types of tissue-specific mtDNA abnormalities–a quantitative loss of mtDNA copy number (mtDNA depletion) and multiple deletions- in humans and in mice. Perturbation of the dNTP pools could also affect the fidelity of mtDNA replication and therefore the quality of the RNA and protein products of the mtDNA. To determine the effect of the reduced dNTP pools on mtDNA fidelity, we performed deep sequencing of purified mtDNA from the livers of two pairs of WT and Mpv17^-/-^ mice. The sequencing coverage was comprehensive for all the samples, with a small trough in the vicinity of the large non-coding region (S4A Fig). The error rates for the wild-type and knockout mice were similar; for one pair, the knockout mouse had a slightly lower error rate than the wild-type littermate (0.033% v 0.043%), and in the other pair a 1.7 fold higher error rate was observed in the knockout mouse (Table 1, run 1). The read depth was lowest in the second knockout animal; however, a replica experiment produced greater depth and confirmed the error rate as higher than the paired control (Table 1, run 2). The error rates for the four individual bases differed to similar extents in all four mtDNA samples (P > 0.05 using one-way ANOVA), with dGTP consistently the lowest (0.007%) and dATP the highest (0.014%). Therefore, the dNTP insufficiency in the Mpv17^-/-^ mouse appears to have little or no effect on the fidelity of mitochondrial DNA replication.

**Table 1:** Mutational load in purified liver mitochondrial DNA of Mpv17^-/-^ mice and controls.

Run	Sample	Total bases	ML (x 10^−4^) all	A	C	G	T
**1**	WT1	87561081	4.3	1.39	1.07	0.82	0.99
**1**	KO2	25034992	3.3	1.00	0.86	0.67	0.77
**1**	WT3	112639285	3.4	1.10	0.78	0.62	0.86
**1**	KO4	5968068	5.8	2.22	1.19	0.82	1.53
**2**	WT3	182010153	9.9	3.19	2.61	1.58	2.36
**2**	KO4	11635983	22.1	7.73	5.07	3.40	5.66

ML—Mutation Load (mean) per site frequency (value 10–4).

KO–Mpv17-/-, WT–wild-type littermates of KO mice.

Individual bases are shown as total number of the mis-incorporated allele divided by total bases (value 10–4).

Imbalances of mitochondrial dNTP pools affect replication fidelity [23] and it has been shown that a greater asymmetry of dNTP levels leads to a higher rate of mutation by the mitochondrial DNA polymerase γ *in vitro* [43,44]. However, rather than being asymmetric, the mitochondrial dNTP pools were close to equimolar in both Mpv17^-/-^ mouse liver and MPV17-deficient human cells, albeit at reduced abundance relative to controls (Figs 2A and 4E and S5). This fits with the hypothesis that the dNTP levels may be reconfigured to the lowest common denominator, and thus are equalized, in order to make the best use of the low dNTP pools and minimize replication infidelity. From this perspective, the two MPV17 disease models are very similar, displaying little or no protein, a dNTP insufficiency adjusted to equimolarity and mtDNA depletion.

### Altered expression of factors involved in mitochondrial dNTP homeostasis in MPV17 deficiency

**Fig 6:**
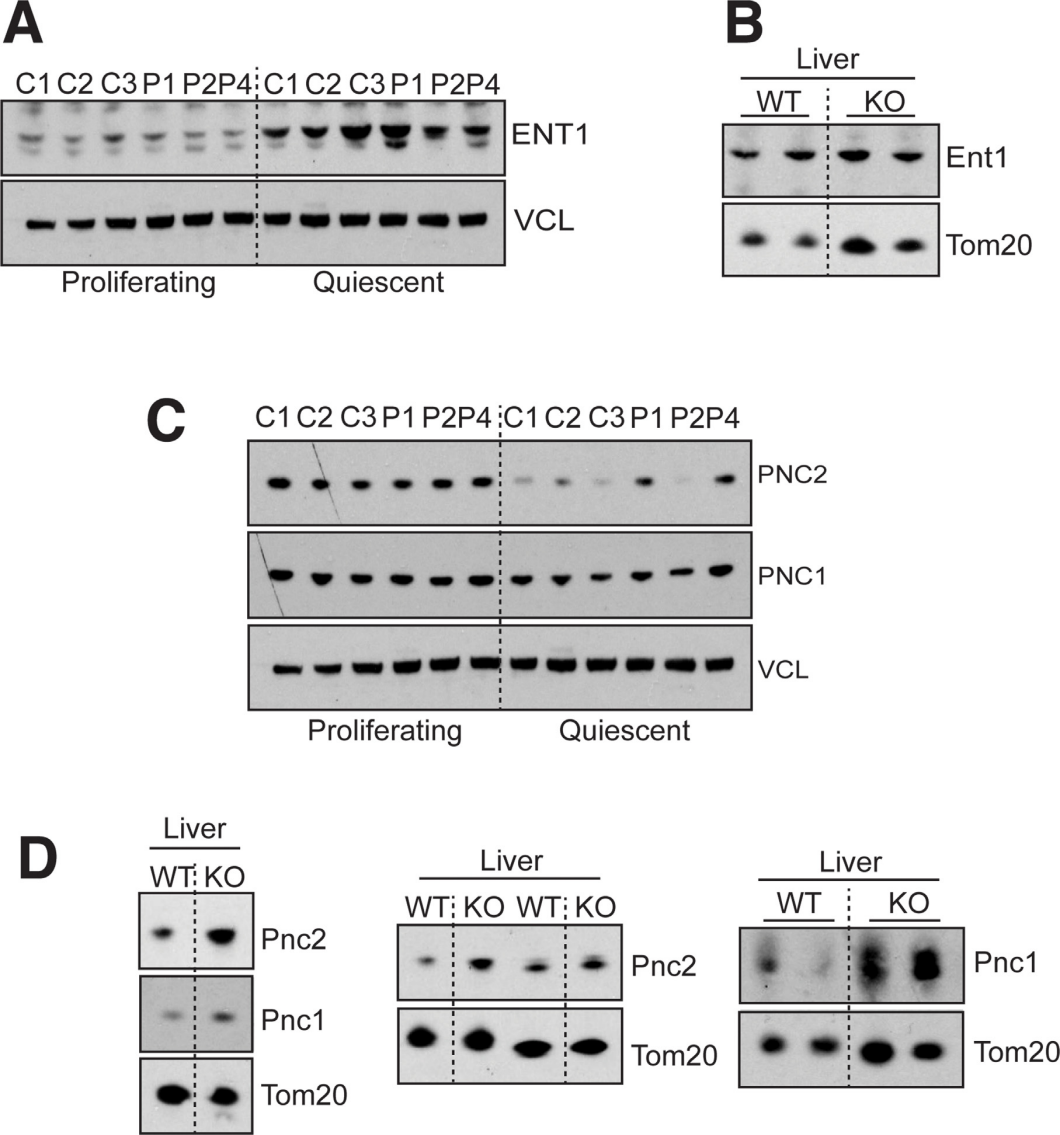
The abundance of the mitochondrial deoxynucleotide (di- and tri- phosphate) transporters is increased in the absence of MPV17. Representative immunoblot of the equilibrative nucleoside transporter (ENT1) in (**A**) control and MPV17-mutant fibroblasts in dividing and quiescent cells, and (**B**) in the liver of wild-type (WT) and knockout (KO) mice. (**C**) Steady state levels of the mitochondrial deoxynucleotide transporters 1 and 2 (PNC1 and PNC2) in control and MPV1*7* deficient fibroblasts in proliferating and quiescent conditions. (**D**) Pnc1 and Pnc2 steady state levels in the liver, of type (WT) and knockout (KO) mice. Vinculin (VCL), and Tom20 were used as loading control.

The dNTP insufficiency resulting from the loss of function of MPV17 could result from an impairment of *de novo* synthesis, the mitochondrial salvage pathway or dNTP (precursor) transport into the organelles. Therefore, we examined the impact of MPV17 loss-of-function on several factors involved in these processes, with the emphasis on the last two pathways in light of MPV17’s inner mitochondrial membrane localization. The equilibrative nucleoside transporter ENT1 is located in the mitochondrial inner membrane [45,46] as well as the plasma membrane, where it supplies the mitochondria with purine and pyrimidine deoxynucleosides for the MSP. Similar to MPV17 and a number of MSP enzymes, its expression is upregulated in control quiescent cells (Fig 6A) and [47]; however, ENT1 protein levels were not affected by MPV17 deficiency in patient-derived fibroblasts (Fig 6A), or tissues of Mpv17^-/-^ mice (Figs 6B and S6A). In contrast to ENT1, the mitochondrial deoxynucleotide (di- and tri- phosphate) transporter PNC2 [48] is repressed in non-dividing cells compared with proliferating cells (Fig 6C). This supports the previously proposed role of PNC2 in mtDNA replication [48]; in cycling cells the cytosolic dNTP pool is high and the mitochondria access most of the precursors of mtDNA synthesis from this pool, with PNC2 acting as a key nucleotide transporter. In quiescent cells, the cytosolic pool shrinks considerably and PNC2’s role diminishes accordingly, which is reflected in its expression (Fig 6C). In the livers of mice lacking Mpv17, Pnc2 expression was elevated (Fig 6D), and it was high in two of three MPV17-deficient cell lines (Fig 6C). Pnc1 levels were also elevated in the liver of Mpv17 knockout mice (Fig 6D), although no change in expression was evident in MPV17 deficient cells (Fig 6C). The tissue-specific increases in the expression of two dNTP transporters in Mpv17 deficiency (Figs 6C and S6A) suggest an attempt by the mitochondria to access more dNTPs from the cytosol in the disease state. This could have two explanations, either MPV17 is a functional substitute for PNC2 (and to a lesser extent PNC1) in non-proliferating cell, promoting cytosolic nucleotide uptake, or it supports the MSP. In the former case the MSP would be expected to be unaffected or elevated, whereas in the latter case deficiencies of the MSP should be evident.

**Fig 7:**
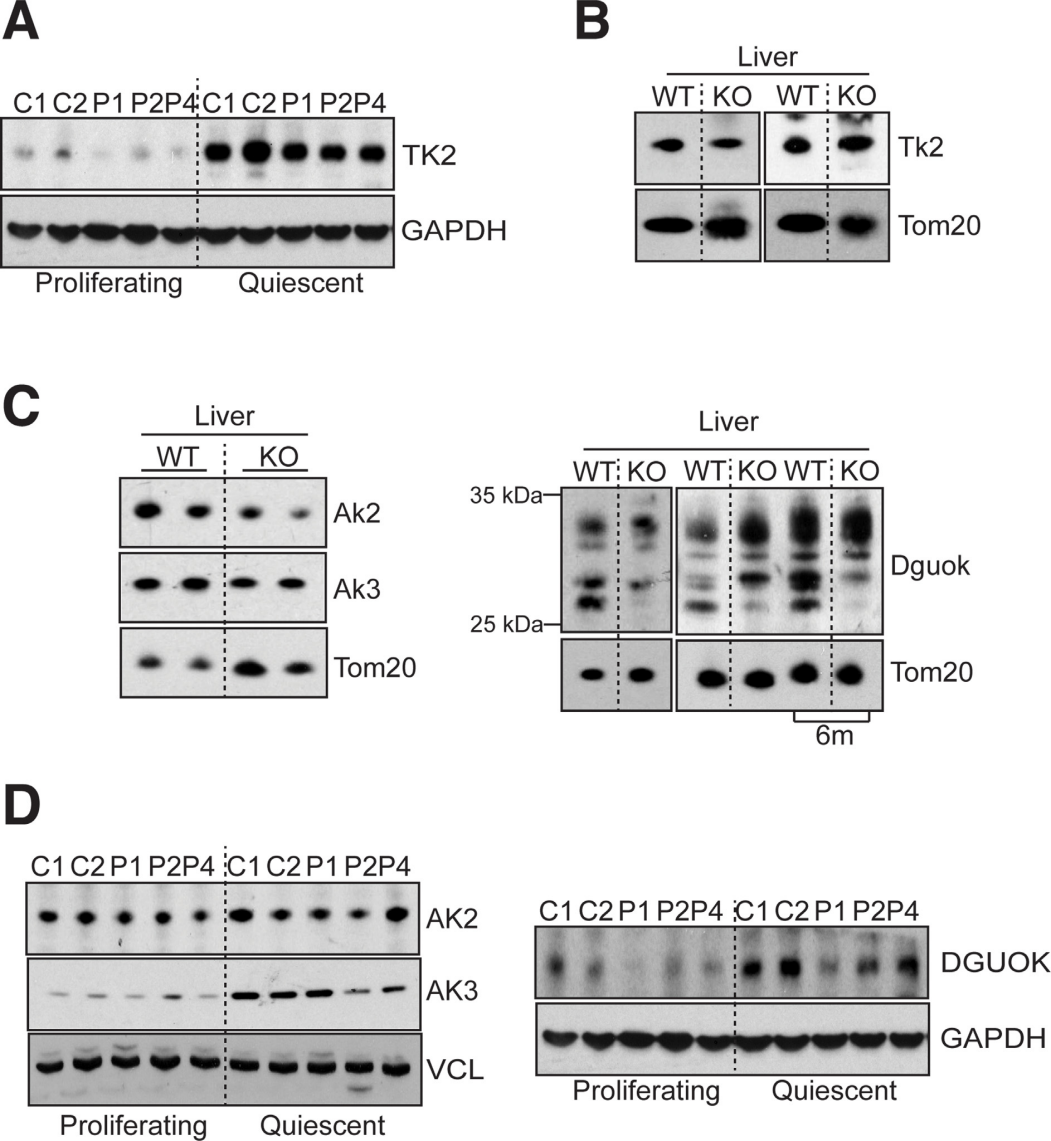
MPV17 loss of function affects the purine branch of mitochondrial salvage pathway. Representative immunoblot thymidine kinase 2 (TK2) in (**A**) control and MPV17-mutant fibroblasts in dividing and quiescent cells, and (**B**) in the liver of wild-type (WT) and knockout (KO) mice. (**C**) Steady state levels of adenylate kinase 2 and 3 (AK2 and AK3) and Deoxyguanosine Kinase (Dguok) in the liver of wild-type (WT) and knockout (KO) mice. The arrow indicates the Dguok isoform downregulated in KO mouse liver. The samples were from 2 month-old mice unless indicated. (**D**) AK2, AK3 and DGUOK steady state levels in control and MPV1*7* mutant fibroblasts in proliferating and quiescent cells. Vinculin, GAPDH, and Tom20 were used as loading control.

The expression of TK2, a key kinase of the pyrimidine branch of the MSP was not affected by the absence of MPV17 either in mutant fibroblasts or mouse tissues (Figs 7A and 7B and S6B). In contrast, analysis of kinases involved in the mitochondrial purine salvage pathway revealed liver-specific decreases of approximately 50% in the amounts of adenylate kinase 2 and 3 (Ak2 and Ak3), in the Mpv17 knockout mouse, and a marked tissue-specific decrease in the expression of an isoform of Dguok (Figs 7C and S6B). These changes in expression suggest that the purine branch of the MSP is repressed in the liver of Mpv17 ablated mice. Furthermore, MPV17 deficiency alters the mitochondrial purine salvage pathway in human fibroblasts that have exited the cell cycle. AK3 protein level was lower than controls in two of three quiescent MPV17 deficient cell lines, and DGUOK was low in all three patient-derived fibroblasts (Fig 7D). The fact that the mutant cells with the highest AK3 expression had the lowest level of DGUOK (Fig 7D, lane 8) suggests the mitochondrial purine salvage pathway is down-regulated in response to MPV17 deficiency by one means or another.

Succinate- CoA Ligase (SUCL) is integral to the citric acid cycle, but is also linked to the MSP via the mitochondrial diphosphate kinase (NDPK) [21,49] and mutations in SUCL are an established cause of MDS [50,51]. Therefore, we also screened Sucla2, Suclg1 and Suclg2 in the three mouse tissues but found no appreciable change in the protein levels between control and Mpv17 knockout mice (S6C Fig).

## Discussion

Although MPV17 deficiency was known to cause mtDNA abnormalities in human, mice and yeast [14,31,52] the basis of the association was unclear. This report shows that loss of function of MPV17 causes nucleotide insufficiency in the mitochondria, thereby establishing the underlying cause of mitochondrial DNA abnormalities in this disease. Hitherto the two main categories of disease gene linked to depletion and multiple deletions of mtDNA encode either components of the replication machinery or enzymes involved in nucleotide metabolism [36]. The clear correlation between MPV17 loss-of-function and nucleotide insufficiency, reported here, places MPV17-related disease firmly in the category of mtDNA disorders caused by deoxynucleotide perturbation, together with mutant forms of *TYMP*, *TK2*, *DGUOK*, *RRM2B* and *ABAT* [2–4,7,21]. Moreover, this report suggests that the pathophysiological consequence of Mpv17 deficiency is a dearth of deoxynucleotides that slows the rate of mtDNA replication, and it is the latter that ultimately causes mtDNA depletion. Thus, it is expected that other cases of nucleotide disturbance will also result in slow mtDNA replication, as previously inferred from the reduced rates of DNA synthesis in an *in organello* model [24]

The marked mitochondrial deoxynucleotide insufficiency resulting from loss-of-function of MPV17 can explain the mtDNA depletion seen in mouse tissues, patient-derived cells, and by inference the patients’ themselves. More specifically, several findings point to perturbed guanosine metabolism as a key feature of MPV17 deficiency. Previously, it was shown that zebrafish deficient in Mpv17 lack stripes owing to an inability to produce guanine crystals [53], the loss of function of deoxyguanosine kinase and MPV17 produce a similar hepatocerebral phenotype in humans [2,14] and dGTP was the dNTP most depleted in Mpv17^-/-^ mouse liver (Fig 2A). The additional findings that MPV17 expression increases in non-dividing cells (Figs 4A and S2C) and enzymes of mitochondrial purine salvage pathway are repressed in MPV17 deficiency (Fig 7C and 7D) support the hypothesis that the purine MSP is dependent on MPV17.

Given MPV17’s location in the inner mitochondrial membrane [14], the recent reports of its channel activity in mammals [54] and in yeasts [55], and the mitochondrial dNTP insufficiency associated with its loss of function (this report), MPV17 might be involved directly or indirectly in the uptake of nucleotides by the mitochondria. Although it would be premature to fully discount this possibility, the pattern in expression of mitochondrial proteins involved in nucleotide metabolism does not support this idea. Thus, while MPV17 might in theory facilitate the action of ENT1 in mitochondrial nucleoside uptake, this role would not account for the repression of mitochondrial salvage pathway enzymes observed in the absence of MPV17. Moreover, ENT1’s expression, which appears to be demand driven in normal [45] and disease states [47], is unaffected by the loss of MPV17. If instead MPV17 were a mitochondrial dNDP/dNTP transporter, we would expect its expression to match that of other proteins involved in the maintenance of mitochondrial dNTP pools in cycling cells, similar to PNC2, whereas MPV17 expression correlates with MSP enzymes and allied proteins. While it could be argued that MPV17 is the replacement for PNC1 and 2 in non-dividing cells, analogous to the p53R2 subunit of RNR, and that PNC1 and PNC2 expression increase in the absence of MPV17 for this reason, this is again difficult to reconcile with a decrease in MSP enzymes. Hence, it is unlikely MPV17 is a deoxynucleotide carrier.

The human PNC1 ortholog in yeasts (rim2) and flies (drim2) makes a critical contribution to the transport of dNTPs into the mitochondria, as evidenced by the loss of mtDNA in both organisms lacking the gene [56,57]. The yeast mtDNA abnormality can be rescued with either PNC1 or PNC2; and human PNC1 and PNC2, and yeast rim2, function as nucleotide transporters in reconstituted liposomes [48,58]. The combination of increases in Pnc2 and Pnc1 expression and decreases in MSP enzymes in Mpv17 deficiency is consistent with the affected mitochondria attempting to increase the import of dNTPs from the cytosol, in response to the dNTP insufficiencies resulting from the restricted MSP. However any increase in flux of deoxynucleotide triphosphates into the mitochondrial matrix is expected to be modest as cytosolic *de novo* synthesis is low in non-dividing cells, and the mtDNA depletion observed indicates the mitochondrial dNTP pools are inadequate for mtDNA replication in liver and non-dividing cultured cells. Finally, there was no evidence of repression of the MSP or increases in PNCs in unaffected tissues, supporting the view that these are critical elements of the MPV17-related MDS.

The changes in the expression of the deoxynucleotide transporters and MSP enzymes also suggest the route by which NdR supplementation rescues and prevents mtDNA depletion in MPV17 deficiency. It is most likely that the exogenous deoxynucleosides are converted to dNTPs in the cytosol and imported into the mitochondria via PNC1 and PNC2, rather than being transported directly into the mitochondria to serve as substrates of the *restricted* MSP. PNC2 in particular could play a critical role in preventing mtDNA depletion: it is the deoxynucleotide transporter upregulated in two of three MPV17 deficient cell lines, it has a higher substrate specificity for dGTP and dCTP than PNC1 [48], and dG and dC proved sufficient to prevent and rescue the mtDNA depletion (Figs 4F and 5C).

The mitochondrial genome has a higher mutation rate than the nuclear genome [59], with explanations ranging from oxidative stress [60] and a limited repair system [61] to dNTP pool asymmetries [43]. The observation that in two models of MPV17 deficiency the pool asymmetry narrows suggests cells and tissues operate to find a balance between replication accuracy and velocity, and that when precursors are scarce, replication fidelity is prioritized. This fits with the model proposed by Mathews and colleagues that equimolar concentrations of the dNTP pools minimize the error rate [43]. Accordingly, while the low dNTPs levels in liver of the Mpv17 KO mouse can sustain mtDNA at only 10% of the normal level, we propose that dNTP equimolarity can maintain mtDNA at this level indefinitely, thereby explaining the early rapid depletion that stabilizes for the reminder of the mouse’s lifespan [31]. The questions as to why mitochondrial dNTP pools are maintained in asymmetry in normal cells, and of how these pools are equalized in the case of MPV17 deficiency, remain to be answered.

Despite the links between MPV17 and guanosine metabolism, GdR was not sufficient to prevent mtDNA depletion in the absence of MPV17, a pyrimidine was also required (Fig 4F). The ‘pool symmetry hypothesis’ offers a possible explanation of this apparent discrepancy: adjustments made in response to the chronic dNTP pool insufficiency cannot rapidly be reversed by an exogenous supply of deoxyguanosine alone. That said, TdR with GdR was no better than GdR alone in preventing mtDNA depletion in the MPV17 mutant cells, presumably owing to the known adverse effects on mtDNA copy number of an excess of thymidine [7,22]. Thymidine supplementation induces mtDNA depletion in cultured cells [62], unless accompanied by deoxycytidine supplementation [24], and here, 50 μM thymidine for 14 days depressed mtDNA copy number by 40% in the control fibroblasts (S3B Fig). Moreover, in isolated organelles, an excess of dTTP impairs mitochondrial DNA synthesis owing to decreased dCTP, irrespective of dGTP abundance [24]. In contrast to TdR, CdR, a precursor of both pyrimidines via the conversion of dCMP to dTMP [20] in addition to GdR prevented and rescued the mtDNA depletion. Thus, the combination CdR plus GdR (with AdR) appears to be the best therapeutic strategy for MPV17 related MDS. The similarities between the two models suggest the benefits of deoxynucleoside supplementation seen in cell culture (Figs 4F and 5B and 5C) might well translate to living organisms, including human patients, as proposed for other MDS caused by dNTP perturbations [63] [40]. Hence, the *Mpv17* ablated mouse represents an important model to evaluate the uptake, therapeutic potential, and possible side-effects of deoxynucleoside administration alone, or in combination with an inhibitor of their catabolism, before contemplating the application of this approach to patients.

## Methods

### Animals and genotyping

Male and female Mpv17-/- CFW embryos were purchased from The Jackson Laboratory (stock number 002208) and bred with MF1 purchased from Charles River, UK. Littermate controls where used for all studies. Animals were genotyped via the polymerase chain reaction using suitable primers (S1 Table). All animal protocols used in this study were approved by the UK Home Office and the University of Cambridge and conducted in collaboration with the Wellcome Trust-MRC Institute of Metabolic Science Disease Model Core

### Cell lines

Primary skin fibroblast cultures were obtained from 4 healthy controls and from patients with mutations in *MPV17* (n = 5) (S2 Table), All cells were negative for mycoplasma based on regular screening using LookOut Mycoplasma PCR Detection Kit (Sigma). Primary fibroblasts were cultured in Dulbecco’s Modified Eagle’s Medium (DMEM, Life Technologies) supplemented with 10% fetal bovine serum (FBS, Hyclone), 1% penicillin and streptomycin (PS, Life Technologies) at 37°C in a 5% CO_2_ atmosphere.

### Mitochondrial isolation and mitochondrial dNTP pool determination

Mitochondria were isolated for mouse tissues (liver, kidney, brain) by differential centrifugation as previously described [14,24]. For dNTP isolation, the mitochondrial pellets were resuspended in MAITE buffer (25mM sucrose, 75mM sorbitol, 100mM KCl, 10 mM H_3_PO_4_, 0.05mM EDTA, 5mM MgCl_2,_ and 10 mM Tris-HCl, pH 7.4), and protein concentration was determined. An aliquot of protein from each mitochondrial preparation (500 μg) was precipitated with 0.5 M trichloroacetic acid (final concentration) by centrifugation at 20,000 x g for 5 min at 4°C, the supernatant was neutralized with 1.5 volumes of 0.5 M trioctylamine in Freon (1,1,2-trichlorotrifluoroethane) and re-centrifuged for 10 min at 10,000 x g at 4°C. The second supernatant was vacuum dried, redissolved in 125 μL of 40 mM Tris-HCl pH 7.4, and stored at -80°C until analysis. Mitochondrial dNTPs were extracted from primary cultured fibroblasts as previously described [62]. dNTP concentrations were determined by a polymerase-based method using mitochondrial extract, as described in [40].

### Immunoblotting

Isolated mitochondria were lysed in 20 mM HEPES, 5 mM EDTA, 75 mM NaCl, 2 mM DTT, 0.4% n-Dodecyl β-D-maltoside (DDM) and 1X protease and phosphatase inhibitor cocktail (Cell signaling and Roche, respectively). Whole cells were lysed in PBS, 1% SDS, 1X protease and phosphatase inhibitor cocktail, and 50 Units benzonase (Millipore). Protein concentration was determined by DC protein assay (Biorad). Western blotting and immunodetection were performed as described in [64]. The primary antibodies and relative dilutions used are described in S3 Table.

### Blue Native Electrophoresis

Isolated mitochondria from mouse liver were resuspended in 1M 6-aminohexanoic acid, 50 mM Bis-Tris-HCl (pH 7.0) at 10 mg/mL final concentration. Mitochondrial membranes were solubilized by the addition of n-Dodecyl β-D-maltoside (DDM) at 1.6 g/g or digitonin (DIG) at 4 g/g. Samples were incubated on ice for 5 min and centrifuged at 16,000 x g for 30 min. Supernatants were collected and combined with an equal volume of native sample buffer (Biorad). Mitochondrial membrane complexes (25 μg) were separated on a NativePAGE 3–12% Bis-Tris gel (Life Technologies) and transferred to PVDF membrane. After blocking, membranes were incubated overnight with the indicated primary antibodies (S3 Table).

### Quantification of mtDNA copy number

Total DNA was isolated from human fibroblasts and mouse tissues using DNeasy Blood and tissues Kit (QIAGEN), according to the manufacturer’s protocol, and quantified by spectrophotometry (Nanodrop, Thermoscientific). Real-time quantitative PCR was performed in triplicates on 96-Well Reaction Plates (Applied Biosystems). Each PCR reaction (final volume 25 μl) contained 25 ng DNA, 12.5 μl of Power SYBR-Green PCR Master Mix (Applied Biosystems) and 0.5 μM of a forward and a reverse primer. MtDNA was amplified using primers specific to a region of murine or human COXII gene and APP1 was amplified as a nuclear gene standard reference. The sequences of the primers used are listed in S1 Table. Changes in mtDNA amount were calculated using the 2^-ΔΔCt^ method [65] and represented as fold changes relative to the indicated control.

### Detection of mitochondrial replication intermediates (mtRIs)

For the analysis of mtRIs, mitochondria were isolated from mouse liver as described above, with an additional sucrose-gradient step to preserve the integrity of the replication intermediates [66]. Nucleic acids were extracted from mouse liver by detergent lysis, protease digestion and successive phenol and chloroform extractions, as described previously [67]. *Bcl*I (New England Biolabs) digestions of mouse liver mtDNA were performed under conditions recommended by the manufacturer. 2D-AGE was performed according to the standard method [68]. After electrophoresis, the DNA was Southern blotted to solid support (Magnaprobe, Osmonics Inc) and the membranes probed with radiolabeled strand-specific RNA probes, generated using T7-maxiscript kit (Ambion) as per the manufacturer’s instructions. The template for the synthesis of an H-strand specific riboprobe corresponding to nt 15,196–16,006 of mouse mtDNA was generated via PCR, using forward and reverse primers, 5´-TAATACGACTCACTATAGG GCCAACTAGCCTCCATCTCATAC-3´ (15,196–15,218, T7 sequence underlined), and 5´-AATGATTCTTCACCGTAGGTGCG-3´ (15,984–16,006), respectively. Hybridizations were overnight at 55°C in 2 x SSPE, 2% Sodium dodecyl sulfate, 5 x Dernhardt’s Reagent, 5% Dextran sulfate buffer. After overnight incubation, membranes were washed 4–6 times with 0.1 x SSPE, 0.5% SDS, at 55°C. Membranes were exposed to phosphorscreens (GE Healthcare) for 12–120 h and imaged on a Typhoon scanner (GE Healthcare).

### Generation of quiescent cultures and transient mtDNA depletion

To obtain quiescent fibroblasts, 2.0 x 10^5^ cells were seeded in 60 mm dishes and grown in 10% FCS until confluent (5–7 days), when the serum was changed to 0.1% dialyzed FBS (Pan Biotech). Where indicated, the medium was supplemented with 50 or 100 μM AdR, CdR, GdR, TdR (Sigma) or different combinations of the four deoxynucleosides. During the treatment, media were replaced every 3–4 days. To obtain quiescent mtDNA-depleted cultures, proliferating fibroblasts were first incubated in DMEM, 10% FCS containing 50 ng/mL ethidium bromide (EB) and 50 μg/mL uridine until confluent (7 days). After a further 7 days of EB treatment in low serum (0.1% dialyzed FCS), EB was removed by washing the cells 5 times, and fresh DMEM with 0.1% dialyzed FCS, 50 μg/mL uridine was added. Quiescent fibroblasts were cultured for a further 14 days, with DNA sampling at intervals. Where indicated, the medium was supplemented with,deoxynucleoside (50 or 100 μM each).

#### Sequencing library preparation and mapping

For the sequencing analysis, mouse liver mtDNA was purified from sucrose-gradient isolated mitochondria as above. Purified mtDNA was fragmented prior to library preparation using a Covaris S220 and the Sonolite software with settings of duty cycle 10%, intensity 5, cycles 200 for 3 min at 4°C. 200 bp paired-end DNA libraries were prepared using the Illumina TruSeq LT kit and run on the MiSeq. We mapped sequencing data (FASTQ) files to the NCBI Reference Sequence: NC_005089.1 assembly of the mouse mitochondrial genome. Reads were mapped using BWA (Burrows-Wheeler Aligner) software [69] version bwa-0.7.8, bwa-mem algorithm with default parameters. The mapped read (sam) files were converted to bam format using samtools version 0.1.19 [70], and the reads sorted and indexed using samtools. Then the two bam files from different sequencing runs for each sample were merged using Picard tool MergeSamFiles. To capture any reads that might have failed the original alignment owing to the circularity of the mitochondrial genome, the mtDNA assembly was cut in half and spliced so that the original start and end positions were juxtaposed, and the mapping and downstream coverage analysis repeated. The highest coverage version of the alignment for each base was taken forward.

#### Calculating coverage depths and mutation loads

The number of single nucleotide substitutions at each individual base and the overall coverage at each base position was calculated using samtools (mpileup). Dividing these numbers by the total read coverage yielded the SNP frequencies for each of the 3 possible mutant alleles, the sum of which gave the total mutation load. ‘Mutation Load’ is likely an over-estimation due to false positives. These can arise from the sequencing technology used and from the complexity of the sequence itself at that locus. However by comparing only samples from within a single sequencing run, the false-positive error rate should remain the same between samples.

### Statistical analysis

Data are expressed as the mean ± standard error of the mean (SEM). Group means were compared using parametric t-test or non-parametric Mann-Whitney test. One-way ANOVA was used to compare more than two independent groups. A *P*-value of <0.05 was considered to be statistically significant.

## Supporting Information

S1 Table List of primers employed in the study. (DOCX)

S2 Table List of the patients and associated gene mutations analyzed in this study. (DOCX)

S3 Table List of antibodies employed in the study. (DOCX)

S1 Fig Interpretation of the pattern of mtDNA replication intermediates in mouse liver. **(A)** Bubble structures are replication intermediates that include the origin, Y–replication fork arc, SY arcs—supra-Ys. and further interpreted in panel (**B**); black lines represent DNA, red lines are RNA, black crosses are indicative of blocked restriction sites. The replicating mtDNA molecules in the Mpv17^-/-^ samples are much more abundant than controls (illustrated in **B**), but show no evidence of increased replication stalling, whose hallmark is the enhancement of origin-containing (bubble) and replication fork (Y) arcs at the expense of the supra-Y arcs (**C)**.
(TIF)

S2 Fig MPV17 and other factors linked to mitochondrial depletion syndromes are upregulated in non-dividing cells. **(A-B**) Steady state levels of R2, TK1, DGUOK, TK2 and p53R2 in proliferating or quiescent control fibroblasts. Proliferating and quiescent panels are directly comparable as they show cropped images from the same blots (samples run on the same gel). (**C**) Representative blot of MPV17 levels in control and MPV1*7* deficient fibroblasts in proliferating and quiescent conditions. (**D**) Steady state levels of MPV17 in control fibroblasts and the five different MPV17 deficient cell lines assessed in this study, in proliferating condition (long exposure).
(TIF)

S3 Fig Deoxynucleoside supplementation prevents the mtDNA depletion in MPV17-deficient fibroblasts. **(A)** Relative mtDNA copy number of quiescent control or MPV17 deficient fibroblasts supplemented with deoxynucleosides. Where indicated fibroblasts were supplemented with 50 μM of AdR, CdR and GdR or AdR, CdR, GdR and TdR. The amount of mtDNA is expressed relative to its amount in proliferating cells (Student’s t test: ***P<0.001). (**B**) Relative mtDNA copy number of quiescent control fibroblasts supplemented with different combinations of deoxynucleosides. Fibroblasts were cultured for 10–14 days in 0.1% dialyzed FCS with or without the deoxynucleoside combination indicated below (50 or 100 μM). The amount of mtDNA was measured by quantitative PCR and expressed relative to its amount in proliferating cells. Note that an excess of thymidine can perturb mtDNA maintenance, unless accompanied by deoxycytidine supplementation, as seen in [1,2].
(TIF)

S4 Fig Mouse mtDNA samples sequence coverage. The mitochondrial genome position (x-axis) versus sequence coverage divided by maximum coverage for each sample. The coverage was calculated using a 2 kilobase sliding window average. MtDNA of the WT and KO samples are indicated, respectively, in black and in red.
(TIF)

S5 Fig Increased dNTP pool symmetry in two models of MPV17 deficiency. Mitochondrial dNTPs levels in mouse liver (left) and quiescent human fibroblasts (right). P values were obtained using Mann-Whitney test (***P<0.001, **P<0.01,*P<0.05, P>0.05—not significant (NS)). The charts are modified from those shown in Figs 2A and 4E.
(TIF)

S6 Fig The expression of several factors involved in nucleotide metabolism is unaffected in brain and kidney of the Mpv17 ablated mouse. Steady state levels of **(A)** Ent1, Pnc1 and Pnc2, (**B**) Tk2, Ak2, Ak3 and Dguok in the brain and kidney of wild-type (WT) and knockout (KO) mice. (**C**) Representative immunoblot of Sucla2, Suclg1 and Suclg2 proteins (the three subunits of Succinate-CoA Ligase) in liver, brain and kidney of the wild-type (WT) and knockout (KO) mice.
(TIF)

S1 References Additional references to the supplemental information. (DOCX)
