# Supplementary material for: MPV17 Loss Causes Deoxynucleotide Insufficiency and Slow DNA Replication in Mitochondria
Source: PLoS Genet. 2016 Jan 13;12(1):e1005779. doi: 10.1371/journal.pgen.1005779 (PMC4711891; doi:10.1371/journal.pgen.1005779)
Supplement: S1 Table — (DOCX) [file pgen.1005779.s001.docx]

- **Table S1. List of all the primers employed throughout the study**

| **Primer name** | **Target** | **Sequence 5’-3’** | **Used for** |
| --- | --- | --- | --- |
| MPVwt/f | *Mpv17* | AACCACTACGGCTGGCTAGA | Genotyping |
| MPVwt/r | *Mpv17* | GCTTCAAAGCAAACGACCTC |  |
| MPVmut/r | *Mpv17^mut^* | CCTACAGGTGGGGTCTTTCA |  |
| Mm-COXII-F | *Cox2* | GAGCAGTCCCCTCCCTAGGA | qPCR |
| Mm-COXII-R | *Cox2* | GGTTTGATGTTACTGTTGCTTGATTT |  |
| Hs-COXII-F | *COX2* | CGTCTGAACTATCCTGCCCG |  |
| Hs-COXII-R | *COX2* | TGGTAAGGGAGGGATCGTTG |  |
| Mm-APP1-F | *App1* | CGGAAACGACGCTCTCATG |  |
| Mm-APP1-R | *App1* | CCAGGCTGAATTCCCCAT |  |
| Hs-APP1-F | *APP1* | TTTTTGTGTGCTCTCCCAGGTCT |  |
| Hs-APP1-R | *APP1* | TGGTCACTGGTTGGTTGGC |  |
