# Supplementary material for: MPV17 Loss Causes Deoxynucleotide Insufficiency and Slow DNA Replication in Mitochondria
Source: PLoS Genet. 2016 Jan 13;12(1):e1005779. doi: 10.1371/journal.pgen.1005779 (PMC4711891; doi:10.1371/journal.pgen.1005779)
Supplement: S2 Table — (DOCX) [file pgen.1005779.s002.docx]

- **Table S2. List of the patients and associated gene mutations analyzed in this study**

| **Patient** | **Gene** | **Mutation** | **Reference** |
| --- | --- | --- | --- |
| P1 | *MPV17* | Homozygous p.(Gln93Pro) mutation | Previously reported in [[3](#_ENREF_3)] as patient 12 |
| P2 | *MPV17* | Homozygous p.(Gln93Pro) mutation | Previously reported in [[3](#_ENREF_3)] as patient 11 |
| P3 | *MPV17* | Compound heterozygous: p.(Lys88Met) and p.(Met89Leu) on one allele; p.(Asp143*) on the other | Previously reported in [[4](#_ENREF_4)] |
| P4 | *MPV17* | Homozygous p.(Asp95fs) mutation | This report |
| P5 | *MPV17* | Homozygous p.(Arg41Trp) mutation | Previously reported in [[3](#_ENREF_3)] as patient 4 |
