# Supplementary material for: MPV17 Loss Causes Deoxynucleotide Insufficiency and Slow DNA Replication in Mitochondria
Source: PLoS Genet. 2016 Jan 13;12(1):e1005779. doi: 10.1371/journal.pgen.1005779 (PMC4711891; doi:10.1371/journal.pgen.1005779)
Supplement: S3 Table — (DOCX) [file pgen.1005779.s003.docx]

**Table S3. List of all antibodies employed throughout the study**

| **Antibody** | **Company** | **Catalog** ♯ | **Dilution** |
| --- | --- | --- | --- |
| anti-MPV17 | Proteintech | 10310-1-AP lot 2 | 1:500 |
| anti-TOM20 | Santa Cruz | sc-11415 | 1:20000 |
| anti-NDUFA9 | Abcam | Ab14713 | 1:2000 |
| anti-NDUFB8 | Abcam | Ab110242 | 1:2000 |
| anti-VDAC1 | Abcam | Ab14734 | 1:4000 |
| anti-SDHB | Abcam | Ab14714 | 1:2000 |
| anti-UQCRC2 | Abcam | Ab14745 | 1:2000 |
| anti-MTCO1 | Abcam | Ab14705 | 1:2000 |
| anti-MTCO2 | Abcam | Ab110258 | 1:2000 |
| anti-COXIV | Abcam | Ab14744 | 1:2000 |
| anti-ATP5A | Abcam | Ab14748 | 1:5000 |
| anti-GAPDH | Sigma | G8795 | 1:30000 |
| anti-TK1 | Abnova | H00007083-M02 | 1:500 |
| anti-TK2 | Gift from Dr Liya Wang | | 1:1000 |
| anti-DGUOK | Gift from Dr Liya Wang | | 1:5000 |
| anti-DGUOK | Sigma | D7320 | 1:500 |
| anti-R2 | Santa Cruz | Sc-10844 | 1:1000 |
| anti-p53R2 | Abcam | Ab8025 | 1:5000 |
| anti-AK2 | Abcam | Ab37594 | 1:1000 |
| anti-AK3 | abcam | Ab119058 | 1:1000 |
| anti-PNC1 | Abcam | Ab97820 | 1:1000 |
| anti-PNC2 | Abcam | Ab154559 | 1:1000 |
| anti-Vinculin | Abcam | Ab18058 | 1:2000 |
| anti-SUCLA2 | Proteintech | 12627-1-AP | 1:2000 |
| anti-SUCLG1 | GeneTex | GTX109215 | 1:20000 |
| anti-SUCLG2 | Abcam | Ab72009 | 1:20000 |
| anti-ENT1 | Lifespan Bioscience | LS-C96639/24938 | 1:1000 |
