# Supplementary figures and images for: MPV17 Loss Causes Deoxynucleotide Insufficiency and Slow DNA Replication in Mitochondria
Source: PLoS Genet. 2016 Jan 13;12(1):e1005779. doi: 10.1371/journal.pgen.1005779 (PMC4711891; doi:10.1371/journal.pgen.1005779)

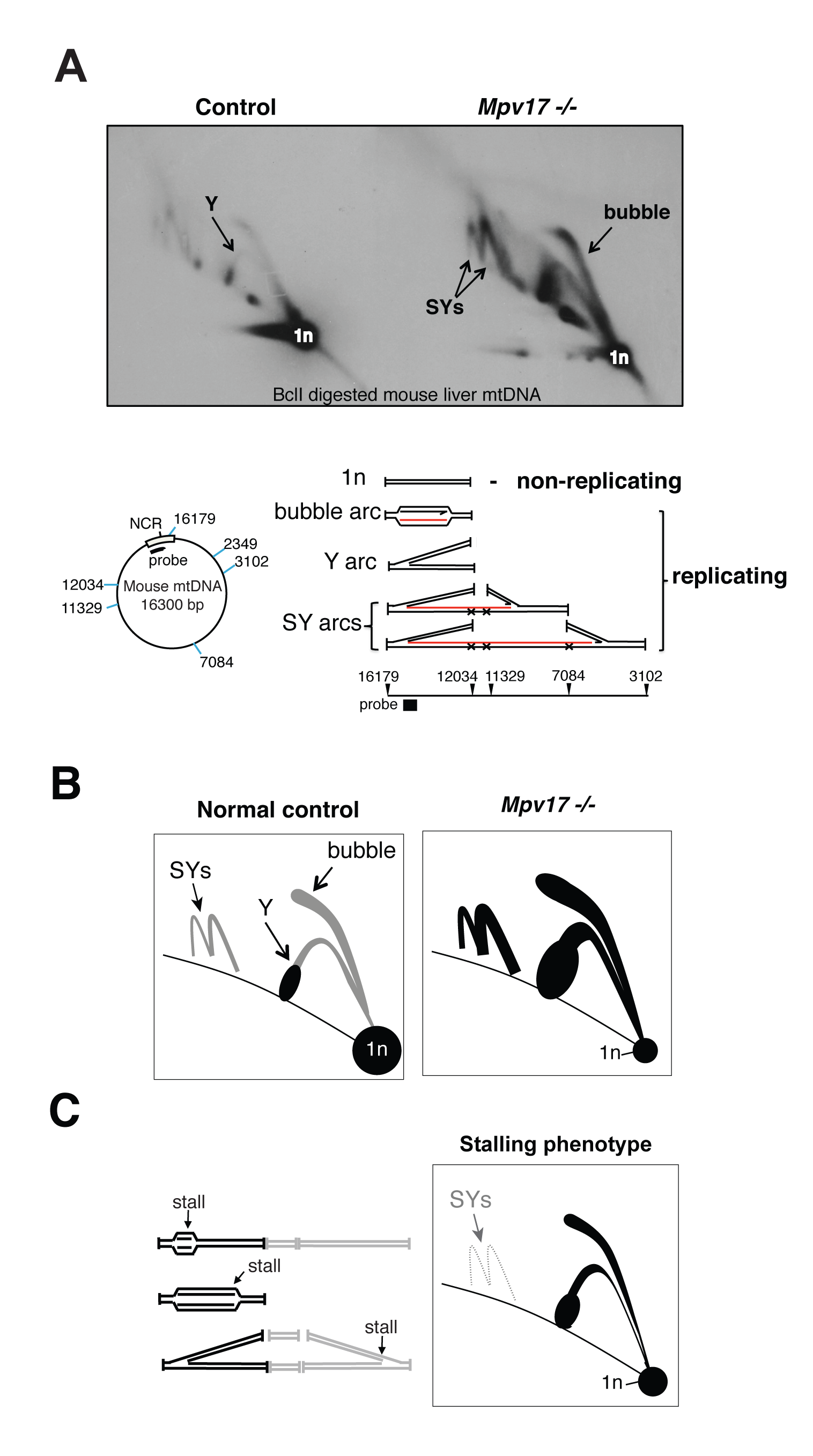

Supplement: S1 Fig — (A) Bubble structures are replication intermediates that include the origin, Y–replication fork arc, SY arcs—supra-Ys. and further interpreted in panel (B); black lines represent DNA, red lines are RNA, black crosses are indicative of blocked restriction sites. The replicating mtDNA molecules in the Mpv17-/- samples are much more abundant than controls (illustrated in B), but show no evidence of increased replication stalling, whose hallmark is the enhancement of origin-containing (bubble) and replication fork (Y) arcs at the expense of the supra-Y arcs (C). (TIF) [file pgen.1005779.s004.tif]

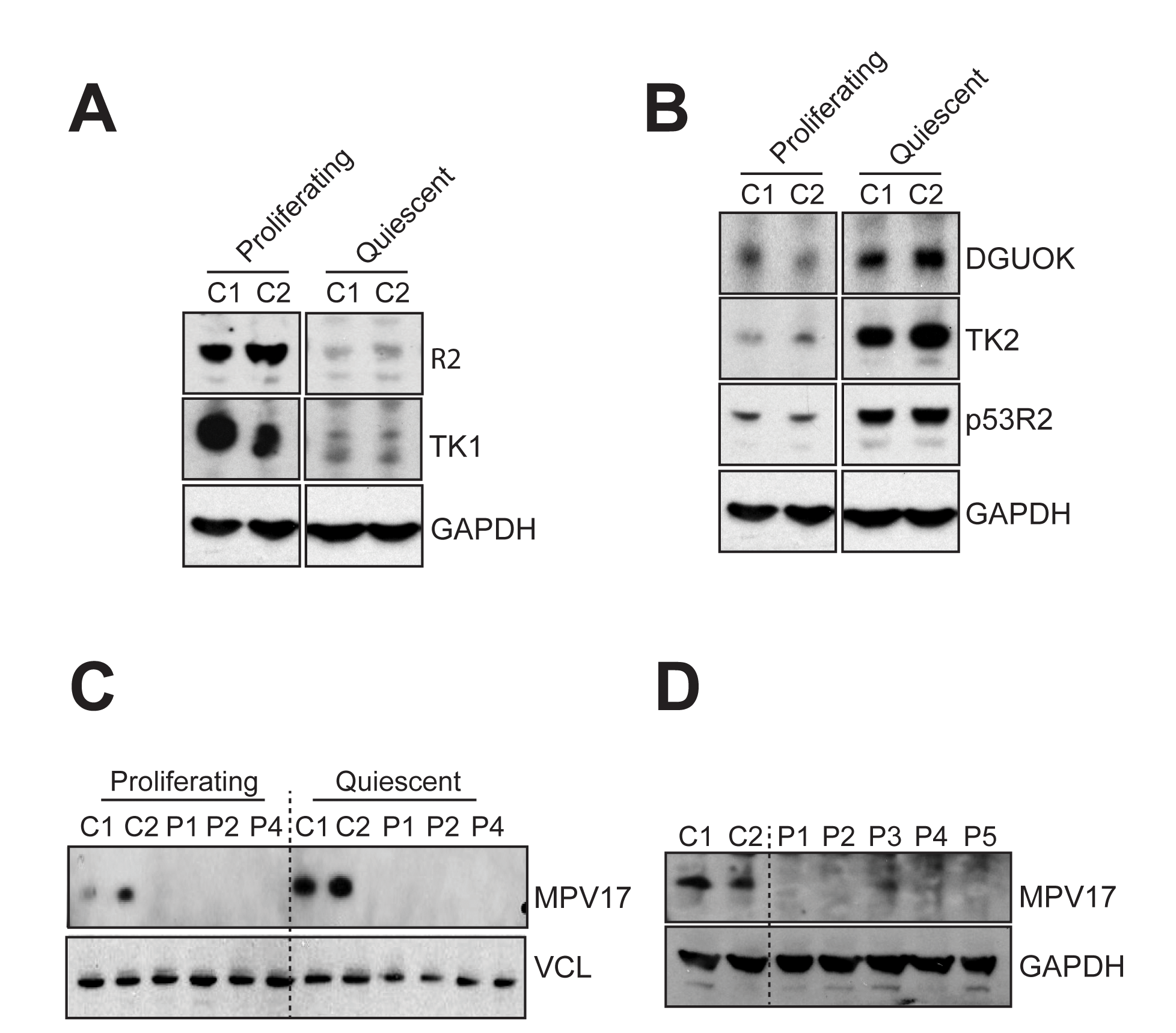

Supplement: S2 Fig — (A-B) Steady state levels of R2, TK1, DGUOK, TK2 and p53R2 in proliferating or quiescent control fibroblasts. Proliferating and quiescent panels are directly comparable as they show cropped images from the same blots (samples run on the same gel). (C) Representative blot of MPV17 levels in control and MPV17 deficient fibroblasts in proliferating and quiescent conditions. (D) Steady state levels of MPV17 in control fibroblasts and the five different MPV17 deficient cell lines assessed in this study, in proliferating condition (long exposure). (TIF) [file pgen.1005779.s005.tif]

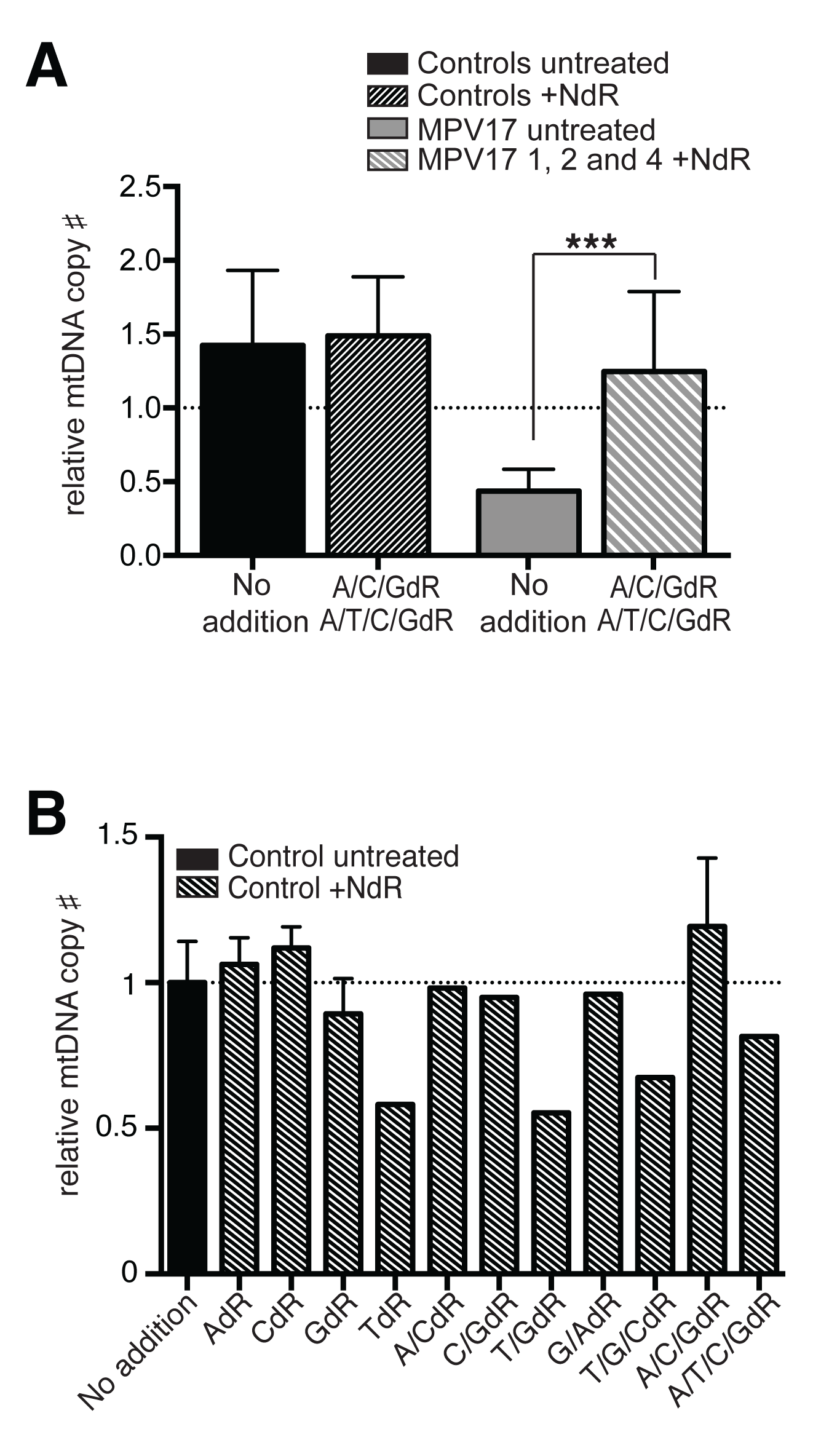

Supplement: S3 Fig — (A) Relative mtDNA copy number of quiescent control or MPV17 deficient fibroblasts supplemented with deoxynucleosides. Where indicated fibroblasts were supplemented with 50 μM of AdR, CdR and GdR or AdR, CdR, GdR and TdR. The amount of mtDNA is expressed relative to its amount in proliferating cells (Student’s t test: ***P<0.001). (B) Relative mtDNA copy number of quiescent control fibroblasts supplemented with different combinations of deoxynucleosides. Fibroblasts were cultured for 10–14 days in 0.1% dialyzed FCS with or without the deoxynucleoside combination indicated below (50 or 100 μM). The amount of mtDNA was measured by quantitative PCR and expressed relative to its amount in proliferating cells. Note that an excess of thymidine can perturb mtDNA maintenance, unless accompanied by deoxycytidine supplementation, as seen in [1,2]. (TIF) [file pgen.1005779.s006.tif]

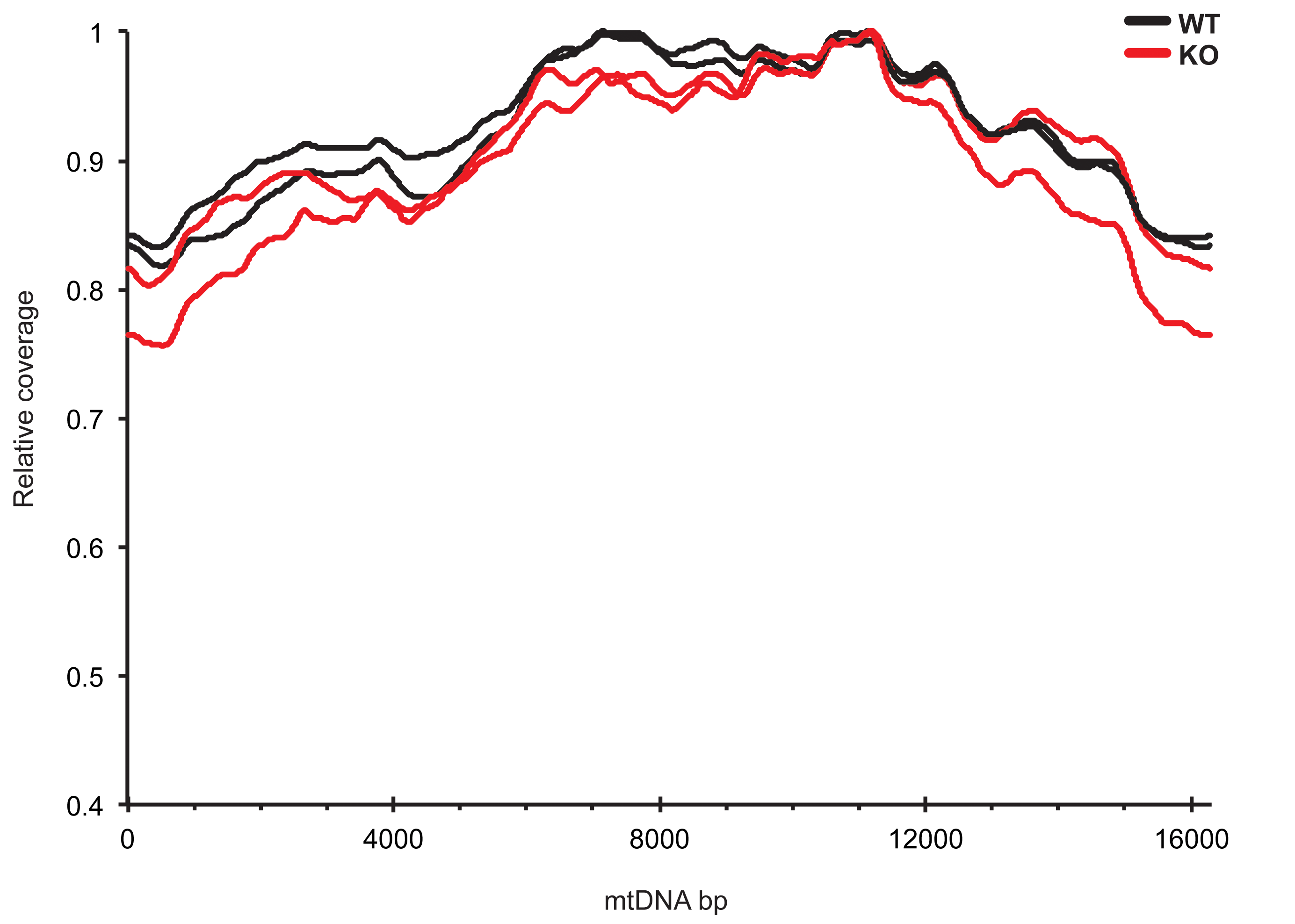

Supplement: S4 Fig — The mitochondrial genome position (x-axis) versus sequence coverage divided by maximum coverage for each sample. The coverage was calculated using a 2 kilobase sliding window average. MtDNA of the WT and KO samples are indicated, respectively, in black and in red. (TIF) [file pgen.1005779.s007.tif]

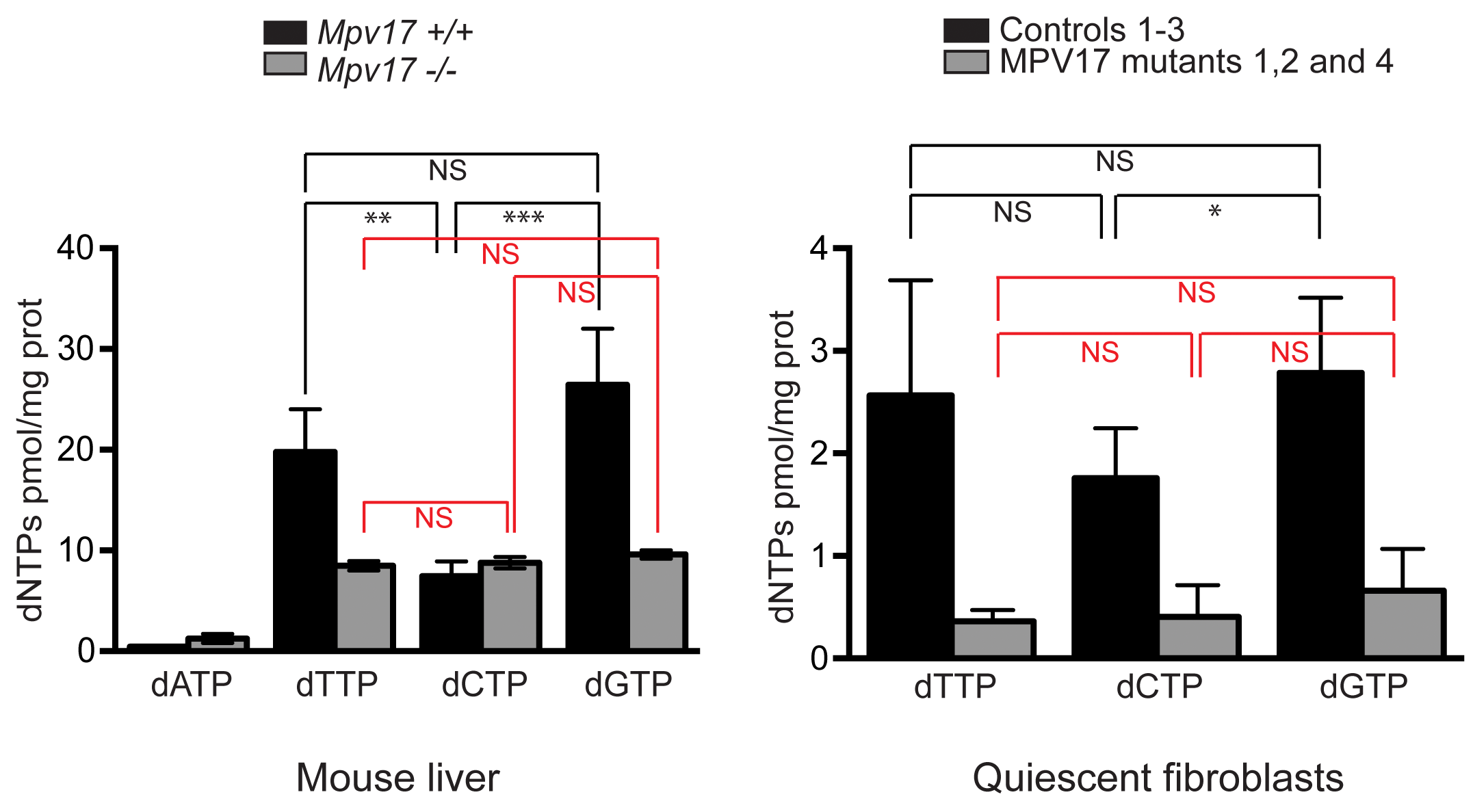

Supplement: S5 Fig — Mitochondrial dNTPs levels in mouse liver (left) and quiescent human fibroblasts (right). P values were obtained using Mann-Whitney test (***P<0.001, **P<0.01,*P<0.05, P>0.05—not significant (NS)). The charts are modified from those shown in Figs 2A and 4E. (TIF) [file pgen.1005779.s008.tif]

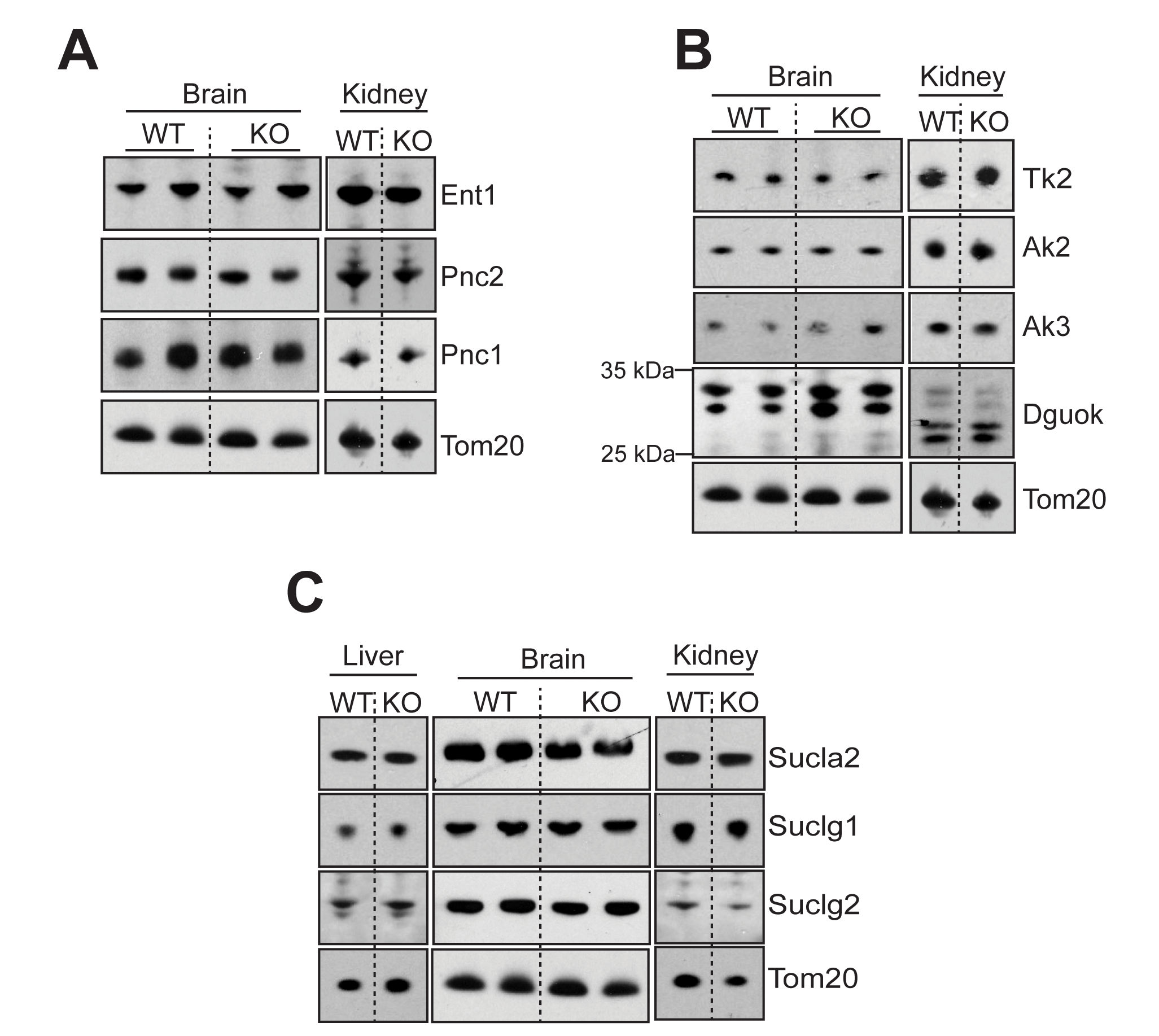

Supplement: S6 Fig — Steady state levels of (A) Ent1, Pnc1 and Pnc2, (B) Tk2, Ak2, Ak3 and Dguok in the brain and kidney of wild-type (WT) and knockout (KO) mice. (C) Representative immunoblot of Sucla2, Suclg1 and Suclg2 proteins (the three subunits of Succinate-CoA Ligase) in liver, brain and kidney of the wild-type (WT) and knockout (KO) mice. (TIF) [file pgen.1005779.s009.tif]
