# Supplementary material for: MPV17 Loss Causes Deoxynucleotide Insufficiency and Slow DNA Replication in Mitochondria
Source: PLoS Genet. 2016 Jan 13;12(1):e1005779. doi: 10.1371/journal.pgen.1005779 (PMC4711891; doi:10.1371/journal.pgen.1005779)
Supplement: S1 References — (DOCX) [file pgen.1005779.s010.docx]

**Supplemental References**

1. Ferraro P, Pontarin G, Crocco L, Fabris S, Reichard P, et al. (2005) Mitochondrial deoxynucleotide pools in quiescent fibroblasts: a possible model for mitochondrial neurogastrointestinal encephalomyopathy (MNGIE). J Biol Chem 280: 24472-24480.

2. Gonzalez-Vioque E, Torres-Torronteras J, Andreu AL, Marti R (2011) Limited dCTP availability accounts for mitochondrial DNA depletion in mitochondrial neurogastrointestinal encephalomyopathy (MNGIE). PLoS Genet 7: e1002035.

3. Uusimaa J, Evans J, Smith C, Butterworth A, Craig K, et al. (2014) Clinical, biochemical, cellular and molecular characterization of mitochondrial DNA depletion syndrome due to novel mutations in the MPV17 gene. Eur J Hum Genet 22: 184-191.

4. Garone C, Rubio JC, Calvo SE, Naini A, Tanji K, et al. (2012) MPV17 Mutations Causing Adult-Onset Multisystemic Disorder With Multiple Mitochondrial DNA Deletions. Arch Neurol 69: 1648-1651.
